# Prioritization of Molecular Targets for Antimalarial Drug Discovery

**DOI:** 10.1021/acsinfecdis.1c00322

**Published:** 2021-09-15

**Authors:** Barbara Forte, Sabine Ottilie, Andrew Plater, Brice Campo, Koen J. Dechering, Francisco Javier Gamo, Daniel E. Goldberg, Eva S. Istvan, Marcus Lee, Amanda K. Lukens, Case W. McNamara, Jacquin C. Niles, John Okombo, Charisse Flerida A. Pasaje, Miles G. Siegel, Dyann Wirth, Susan Wyllie, David A. Fidock, Beatriz Baragaña, Elizabeth A. Winzeler, Ian H. Gilbert

**Affiliations:** † Wellcome Centre for Anti-Infectives Research, Division of Biological Chemistry and Drug Discovery, University of Dundee, Dundee, DD1 5EH, United Kingdom; ‡ Department of Pediatrics, School of Medicine, University of California, San Diego, La Jolla, California 92093, United States; § Medicines for Malaria Venture, 1215 Geneva, Switzerland; ∥ TropIQ Health Sciences, 6534 AT, Nijmegen, The Netherlands; ⊥ Global Health, GSK, 28760-Tres Cantos, Madrid, Spain; # Division of Infectious Diseases, Department of Medicine and Department of Molecular Microbiology, Washington University School of Medicine, St. Louis, Missouri 63110, United States; ∇ Wellcome Sanger Institute, Wellcome Genome Campus, Hinxton, CB10 1SA, United Kingdom; ○ Infectious Disease and Microbiome Program, Broad Institute, Cambridge, Massachusetts 02142, United States; ◆ Department of Immunology and Infectious Diseases, Harvard T.H. Chan School of Public Health, Boston, Massachusetts 02115, United States; ¶ Calibr, a Division of The Scripps Research Institute, 11119 North Torrey Pines Road, La Jolla, California 92037, United States; □ Department of Biological Engineering, Massachusetts Institute of Technology (MIT), Cambridge Massachusetts 02139-4307, United States; % Department of Microbiology and Immunology, Columbia University Irving Medical Center, New York, New York 10032, United States; & Lgenia, Inc., Fortville, Indiana 46040, United States; + Division of Infectious Diseases, Department of Medicine, Columbia University Irving Medical Center, New York, New York 10032, United States

**Keywords:** malaria, Plasmodium, drug discovery, molecular targets

## Abstract

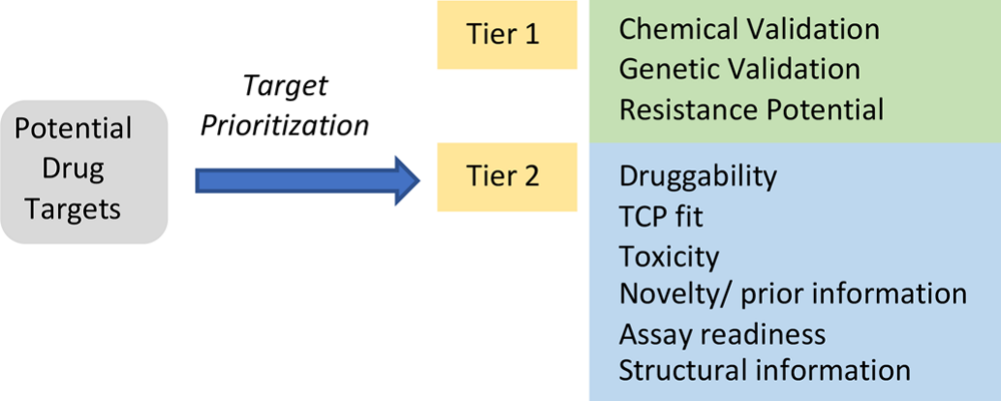

There is a shift in antimalarial drug discovery from phenotypic screening toward target-based approaches, as more potential drug targets are being validated in *Plasmodium* species. Given the high attrition rate and high cost of drug discovery, it is important to select the targets most likely to deliver progressible drug candidates. In this paper, we describe the criteria that we consider important for selecting targets for antimalarial drug discovery. We describe the analysis of a number of drug targets in the Malaria Drug Accelerator (MalDA) pipeline, which has allowed us to prioritize targets that are ready to enter the drug discovery process. This selection process has also highlighted where additional data are required to inform target progression or deprioritization of other targets. Finally, we comment on how additional drug targets may be identified.

Malaria is caused by *Anopheles* mosquito-transmitted protozoan *Plasmodium* parasites. Five species of *Plasmodium* parasites cause human disease: *Plasmodium falciparum*, *P. vivax*, *P. ovale*, *P. malariae*, and *P. knowlesi*. Of these, *P. falciparum* is associated with the most deaths, although infections due to *P. vivax* and *P. knowlesi* can cause severe disease. Infection with *P. vivax* and *P. ovale* is associated with dormant liver-stage forms (hypnozoites) that can be activated months to years after the primary infection, leading to relapse and recurring disease.

Malaria remains a major threat to human health, imposing a heavy social and economic burden, particularly for people from low- and middle-income countries. An estimated 229 million new cases occurred in 2019, predominantly in sub-Saharan Africa, South America, and Asia, resulting in an estimated 409 000 deaths.^1^ Children under five and pregnant women are at higher risk of suffering from severe malaria that can lead to death.

According to the WHO 2020 report on malaria, annual mortality decreased by 60% over the period 2000 to 2019, an unprecedented level of success that saved an estimated 7.6 million lives. However, despite the considerable progress made, these gains have plateaued since 2015 and the Global Technical Strategy (GTS) goals for reductions of at least 40% compared to 2015 in both disease and death by 2020 have been missed.^1^ Since 2000, a variety of interventions have helped to drive this progress, including the use of more extensive vector control measures, reliable diagnostic tests, and improved drug therapy. However, continued progress is hampered by the emergence of mosquitoes resistant to current insecticides and parasites resistant to currently used antimalarial drugs, especially in the Greater Mekong Subregion.^2^ Thus, continued investment in research and development is needed to develop the tools required to stay ahead of resistance and generate the progress needed to achieve the GTS goals.

History has demonstrated that *P. falciparum* resistance to widely used antimalarials inevitably emerges, limiting their effectiveness. There is established resistance or partial resistance to nearly all currently registered antimalarial drugs (with the exceptions of lumefantrine and pyronaridine), and the need for new drugs working through differentiated modes of action is urgent. The emergence of resistance can be delayed by combining antimalarials with different modes of action. Therefore, the identification of new, validated drug targets is crucial in developing novel antimalarials capable of treating parasite populations resistant to current therapies.

The challenge is to identify new treatments that are well-tolerated in vulnerable populations, such as pregnant women, children under 5, and people suffering from malnutrition or coinfection with other pathogens. Another challenge is how best to treat people with asymptomatic malaria and protect vulnerable populations in endemic regions from becoming symptomatic.^3^ To guide the discovery of new treatments that tackle these challenges, clear Target Product Profiles (TPP; descriptions of medicines) are required. These are then used to derive Target Candidate Profiles (TCP; descriptions of molecules) to guide the antimalarial drug discovery and development process. In 2017, Medicines for Malaria Venture (MMV), in discussions with the wider malaria community, updated the TCPs (Table 1) and TPPs (Table 2)^4^ defined first in 2013,^5^ taking into account the learnings and insights gained in recent years. With the goal of eradicating and not just controlling malaria, the new drug pipeline should contain, in addition to compounds efficacious enough to treat symptomatic malaria, drugs able to (1) interrupt disease transmission; (2) prevent relapsing malaria, due to hypnozoites;^6,7^ (3) protect the most vulnerable patients from getting disease; and (4) clear the malaria burden by treating asymptomatic cases of malaria.

**Table 1:** Target Candidate Profile (TCP) definitions

TCP	Goal	Definition
TCP-1	Treatment of disease (both severe and uncomplicated) and chemoprophylaxis (protecting vulnerable populations)	Compounds active against the asexual blood stage of the *Plasmodium* life cycle and active against all resistant strains
TCP-3	Anti-relapse (treatment for recurrent malaria)	Compounds active against liver stage hypnozoites
TCP-4	Prophylaxis (for migratory population or outbreak prevention)	Compounds active against liver stages (ideally providing protection for at least a month)
TCP-5	Transmission blockers (prevention strategies, e.g., treatment of asymptomatic infection)	Compounds active against parasite gametocytes
TCP-6	Transmission blockers	Compounds that block transmission by targeting the insect vector (mosquitocides / endectocides)

**Table 2:** Target Product Profile (TPP) definitions

TPP	Goal	Definition
TPP-1	Treating active disease	Ideally, a combination of TCP-1 with TCP-5 or TCP-3 in order to cure acute or uncomplicated malaria in both adults and children, ideally given as a single oral dose. A fast-killing TCP-1 compound with parenteral administration is essential for severe malaria.
TPP-2	Chemoprotection	Ideally a combination of TCP-4 and TCP-1 (for emerging infection) with the goal to treat migratory populations or prevent outbreaks.

Phenotypic screening platforms focusing on different life cycle stages of the parasite have been developed that can identify hits with antimalarial activity.^8−12^ Targets associated with phenotypic hits are rarely identified during the early stages of drug discovery, which could prove a challenge to the downstream development and optimization of these compounds. Target-based drug discovery can offer several advantages over traditional phenotypic screening:

• The ability to develop target-specific biochemical assays, allowing the identification of chemical start points of insufficient potency to be found in a phenotypic screen.

• The possibility for alternative hit generation approaches such as fragment and structure-based methods, virtual screening, or screening of DNA-encoded libraries (DEL).^13^

• Utilization of structural information to design selectivity against the human orthologue, when present.

• Facilitate scaffold hopping, in the event of pharmacokinetic or toxicological issues associated with a particular chemical series.

• Structure-based approaches have also been used to develop compounds with reduced potential for resistance to emerge.^14^

• Knowledge of the target is also important in designing combination treatments.

• Knowledge of the target is useful in monitoring for resistance development during clinical trials and following deployment.

MalDA (Malaria Drug Accelerator) is an international consortium of 17 groups funded by the Bill & Melinda Gates Foundation. The goal of MalDA is to identify novel drug targets in *Plasmodium*, primarily by linking active compounds to molecular targets through comprehensive mode of action studies.^15^ More recently, the consortium’s mission has expanded to include the development of target-specific assays, hit discovery, and compound progression to early lead status. The early lead criteria that we are adopting are those defined by MMV. This is presented on their Web site (www.mmv.org). MalDA members are summarized in Figure 1.

**Figure 1:**
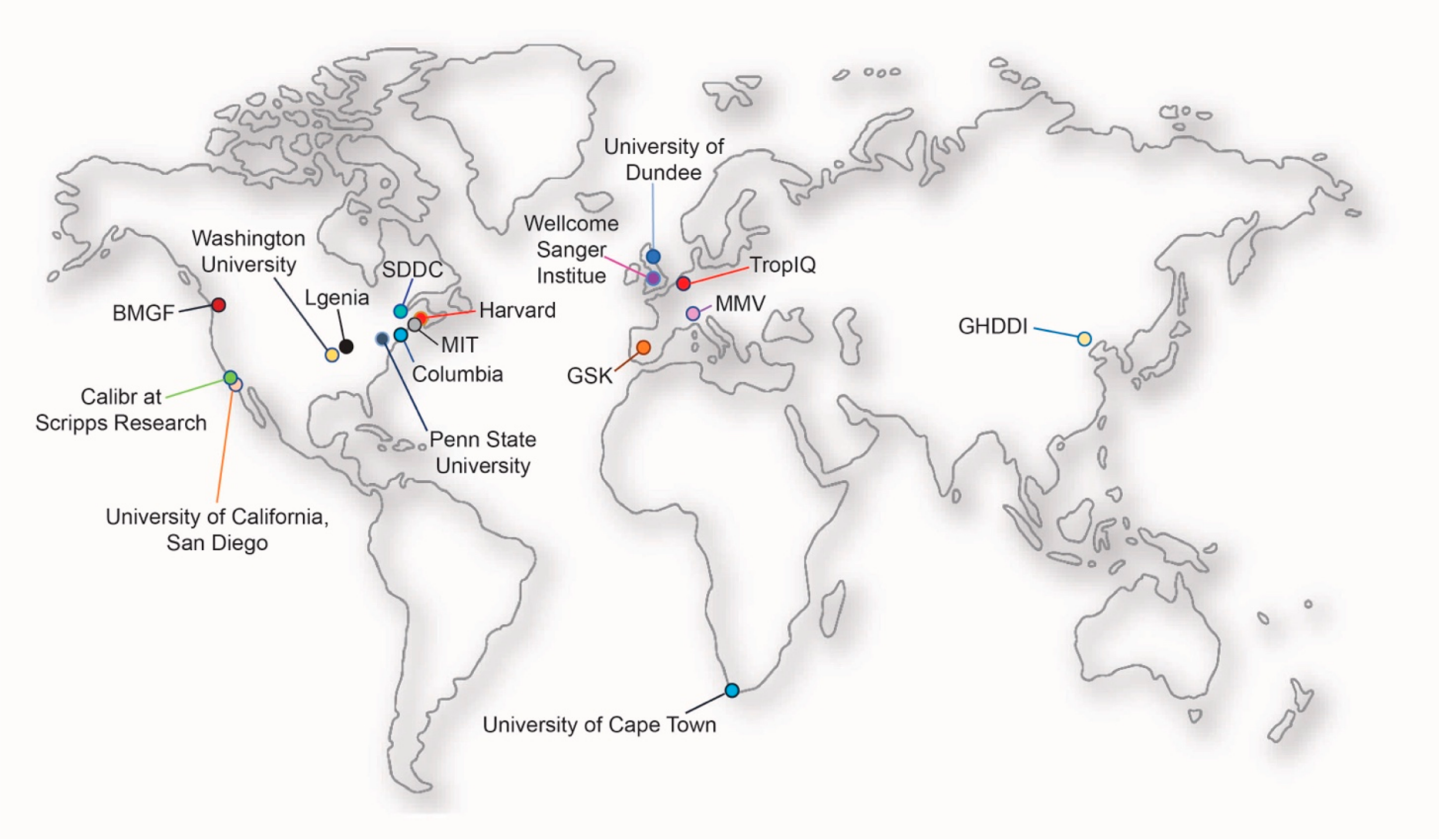
Geographical location of MalDA consortium members. MalDA, with its state-of-the-art *Plasmodium*-adapted technology platforms in bioinformatics, chemo-informatics, chemo-proteomics, genetic manipulation, metabolomics, *in vivo* resistance evaluation, and medicinal chemistry expertise, is at the forefront of the antimalarial drug discovery process by providing tools to accelerate the finding of new starting points for drug discovery (www.malariaDA.org).^15^

## How Are Tractable Drug Targets Identified?

Tractable antimalarial targets can be identified from several different sources. First, they can be found by establishing the molecular targets of phenotypic screening hits of *in vitro* cultured *P. falciparum* asexual blood stage parasites. Target deconvolution is carried out using a variety of approaches. The principal route has involved *in vitro* resistance generation followed by whole-genome sequencing or previously by whole-genome microarray analysis.^15,16^ This strategy has proven highly successful in identifying the molecular targets of many, but not all, phenotypically active compounds.^17−19^ The location of the mutations can give indications as to whether and where the compound binds to the protein. Indeed, this approach can provide important additional information such as identifying and understanding mechanisms of resistance associated with the target. While mechanisms of resistance can give indications of the mode of action, they can also occur in general resistance mechanisms, such as efflux pumps or other modulators of drug potency.

Additional techniques are now being utilized to identify the mechanisms of action of phenotypic actives, which are particularly valuable when resistant parasites cannot be selected. One of these, Thermal Proteome Profiling,^20^ is a mass-spectrometry-facilitated approach that exploits the biophysical principle that binding of a ligand induces thermal stabilization of target proteins. Shifts in the thermal stability of proteins within the parasite proteome are monitored in the presence and absence of drug to identify putative targets. Another technique, metabolomics, is used to give a broad indication of metabolic pathways affected by drug action and provides metabolic fingerprints of compounds acting via previously defined modes of action.^16^

Once the target(s) of phenotypic hits is(are) identified by these primary methodologies, secondary experiments are required to validate this(these) putative target(s). Despite the increasingly sophisticated battery of techniques available to identify molecular targets, there are still compounds where the target or mode of action has yet to be established. This may be due to factors such as compounds working through polypharmacology or targeting host proteins or targets for which it is really difficult to raise resistance. Additionally, some compounds also act via interaction with nonprotein targets, which can be challenging to deconvolute.

Several targets with potential to be exploited for drug discovery have also been suggested by literature precedent, or through general precedence as drug targets in other disease areas. Currently, there is considerable interest in amino acyl tRNA synthetases as potential antimalarial targets. These enzymes have been established as viable drug targets in a number of different pathogens. Interestingly, a number of these enzymes have been found to be the target of phenotypic hits in *Plasmodium* using target deconvolution methods described above.^21−23^ Literature-based targets, however, need careful scrutiny since there can be a disconnect between activity against a molecular target in a biochemical assay and activity in cellular and animal models of disease.^24^ Indeed, the failure of biochemical/enzymatic hits to translate to growth inhibition and cytocidal activity against the parasite has been a confounding factor in the identification of lead compounds, not only for antimalarials but for antimicrobials in general.

Finally, fundamental research by the malaria research community, both within and outside MalDA, has led to the identification of numerous potential drug targets. Once again, targets identified via this route require careful assessment prior to embarking on expensive and time-consuming drug discovery programs.

## Overview of Requirements for a Drug Target

When pursuing a target prioritization program, it is important to identify the key requirements for a drug target. We^25−28^ and others^29^ have published articles in this area. The target must be essential for the progression or pathophysiology of the disease; in the case of *Plasmodium*, this equates to survival or transmission of the parasite. The target also needs to be druggable: its function needs to be modifiable by a small molecule or biologic. In the case of malaria, this must be an orally available small molecule, to meet the criterion of a low cost of goods. Another important concept is *target vulnerability*; this is how much and for how long it is necessary to modulate the activity of the target to have the desired phenotypic effect. It is very challenging to maintain high levels of pharmacological inhibition of a target for prolonged periods of time; therefore, targets requiring relatively low levels of inhibition and/or a short duration of action to quickly “tip” the parasites to death are more attractive. Genetic approaches to engineer the conditional knockdown of a desired target are important here.^30,31^ Further, in the case of malaria, the rate of parasite kill is vitally important, since patients with malaria can rapidly become severely ill and die. Therefore, targets that require inhibition for prolonged periods to kill a parasite should not be pursued to treat asexual blood stage infections. For prophylaxis or transmission blocking, a slower rate of kill may be acceptable.

Another important consideration is the emergence of drug resistance, which is a major concern for anti-infectives, including antimalarials. A target with low resistance propensity, or no obvious bypass mechanism, is preferable. However, understanding resistance mechanisms could lead to alternate treatments (e.g., combination therapy) or attempts to redesign compounds, for example to mitigate the impact of a commonly occurring resistance mutation.^32^ Analysis of existing polymorphisms (using genomic databases of thousands of patient isolates) could also be a consideration. A high level of polymorphism could be indicative of higher flexibility to accumulate protein changes and thus an increased propensity to select resistance.

Selectivity is also a requirement, aiming to minimize off-target liabilities that might cause drug candidates to fail later in development. For malaria, compounds should selectively modulate the parasite target over the host equivalent, unless there is a biological reason why inhibition of the host equivalent does not lead to toxicological consequences. The latter is very difficult to predict however. Since most drug discovery paradigms use some form of screening (usually high-throughput) to identify chemical starting points, there also needs to be a route to develop an appropriate assay for a drug target. While not considered a prerequisite for choosing a target, having access to structural information can be a major benefit, as described above.

Some requirements are specific to malaria and must be met for a particular target to be viable (see Table 1). For example, several species of *Plasmodium* cause malaria, and having a target that is conserved in these species enhances the likelihood that a single treatment can be developed. There are also different life cycle stages for the parasite, so having a compound that can act at multiple stages is desirable.

## Process for Target Prioritization

MalDA has a large portfolio of potential drug targets. We decided to carry out a target prioritization process for several reasons: (1) to identify targets that have the potential to progress into drug discovery programs; (2) to identify targets lacking key information or validation required for progression; (3) to efficiently prioritize future work; and (4) to disseminate our assessment of targets more broadly to the malaria drug discovery community. To facilitate this process, we have generated “target cards” where the information for each criterion is stored in summary form for each of the targets.

We designed a two-tier cascade, associated with a ranking system to assess and prioritize targets (Table 3) based on previously published concepts.^25−29^ In tier 1, the level of genetic and chemical validation and the potential to develop resistance were evaluated, while tier 2 encompassed druggability, TCP fit, toxicity, novelty/prior information, assay readiness and structural information. The scoring system, while not perfect, is a way of highlighting high-priority targets. Certain criteria are stop/go decision points. For example, a target that is not essential or does not fulfill one specific TPP/TCP should not progress into drug discovery.

**Table 3:** Criteria for Target Prioritization

tier 1 target assessment		ranking
Genetic validation	Conditional knockout. Target vulnerability upon conditional knock-down	high
	Essential in genome-wide saturation mutagenesis in *P. falciparum* and/or homologous recombination-mediated knockout screen in *P. berghei*	medium
Chemical validation	Compound-target pair established rigorously	high
	Good correlation between enzyme and cell activity over 3 log units for a compound series	medium
Resistance potential	Irresistible—no resistance found in selections	high
	MIR 8–9 and no cross resistance with any drug in clinical use or development	medium
	6 < MIR < 8 and no cross resistance with any drug in clinical use or development	low
	MIR ≤ 6 and an EC_50_ shift > 10-fold; or evidence of high-grade resistance-conferring SNPs in field isolates; or enzyme not conserved across *Plasmodium* species	STOP

tier 2 target assessment		score
Druggability	Identification of small molecule inhibitors with drug-like physicochemical properties	high
	Computational analysis of the crystal structure or a high quality homology model	medium
TPP/TCP fit	Must be active against at least two life cycle stages at similar concentrations OR blood stage asexual stages with a fast rate of kill	STOP/GO
Toxicity	No close orthologue present; selective small molecule inhibitors for parasite enzyme vs human enzyme	high
Novelty/prior information	Previous work has indicated issues with chemistry, but potential way forward using new information/chemistry	medium
	Previous work has indicated that drug-like inhibitors with *in vivo* activity can be generated and compound(s) in late stage development; potential for back up compound.	low
Assay readiness	Protein expressed and biochemical assay developed for this protein or a close orthologue	high
	No *P. falciparum* protein expressed, but (evidence for) assay for orthologue or reporter cell assay developed	medium
Structural information	Structure of target protein and cocrystal structures with ligands; evidence that it can be soaked	high
	Structure of target protein, but no complex; close orthologue with structures; potential for chimeras	medium

### Tier 1

We considered three different genetic validation methods to determine the essentiality of a *Plasmodium* gene. First, the genome-wide saturation mutagenesis screen in *P. falciparum* asexual blood stages identified likely dispensable and essential genes through mutagenesis index scores (MIS) and mutagenesis fitness scores (MFS).^33^ Second, homologous recombination-mediated knockout screens in the rodent malaria parasite *P. berghei* provide additional information on likely essential genes.^34^ Finally, validation of the genetic essentiality of some targets of interest was available from conditional knockouts and knockdown experiments carried out mostly by the Niles lab.^30^ In this context, conditional knockdowns are of interest to assess target vulnerability, i.e., the duration and extent of reduction of target levels or activity required to compromise parasite viability. In addition, the presence of loss-of-function alleles in the gene in whole-genome sequences from either population studies or *in vitro* resistance selection experiments were also considered as risks. Molecular targets scored high when the corresponding gene was deemed to be essential by the three methods. A target was considered to have no potential for drug discovery if viable parasites were obtained when the gene was abolished completely in a knockout experiment.

Two different levels of chemical validation are considered in our assessment, with the highest score for chemical validation being given to compound–target pairs that have been rigorously established. Ideally, this should include reduced compound susceptibility of transgenic parasites that encode the mutations selected through drug pressuring studies and evidence of compound inhibition of recombinant enzyme. Targets showing good correlation between recombinant enzyme inhibition and parasite growth inhibition over 3 log units for a compound series were also scored for chemical validation.

The final criterion in tier 1 is the resistance potential. First, targets associated to compounds showing a Minimum Inoculum for Resistance (MIR) > 8 (i.e., requiring a minimum of 10^8^ parasites to generate resistance) are preferable and received the highest score, while those with a MIR ≤ 6 with a >10-fold increase in EC_50_ were considered high risk and were not recommended for drug discovery projects.^35,36^ In addition, the MalariaGen database provides genome variation data on over 7000 *P. falciparum* genomes, allowing us to search for the number of SNPs, amino acid changes, and known resistance mutations for a gene of interest (www.malariagen.net).^37^ Preferably, a target would have a high degree of conservation across field isolates and across multiple *Plasmodium* species.

Targets scoring well for essentiality, for parasite survival, and with a manageable resistance risk can progress to tier 2 assessment.

### Tier 2

Molecular targets with known small molecule inhibitors with drug-like properties^38−40^ receive the highest possible score for druggability. In the absence of known inhibitors, computational analysis of the crystal structure or a high-quality homology model can also be used to assess druggability.^41^ Some target classes have been extensively investigated in the context of drug discovery in other disease areas and therefore are expected to be druggable, for example, kinases or bromodomains. For targets for which there is prior drug discovery experience, it is important to leverage prior knowledge. For those programs that have been terminated, was this due to a fundamental issue of target biology? Or was the chemical matter inappropriate, and if so, what is the likelihood of overcoming this? If there is a drug discovery program ongoing elsewhere, is there something different that can be added? It is important not to duplicate work, particularly in a resource-limited disease area. However, given the high attrition rate, until a compound has reached clinical proof of concept, backup strategies need to be considered.

The tier 2 assessment includes an evaluation of the potential to develop selective inhibitors for the targets of interest. The absence of a close human orthologue or the presence of known selective inhibitors are the best indicators. When there is a human orthologue and no known selective inhibitors, a promising target should at least show structural evidence that there are exploitable differences in active sites of the parasite and the human orthologue to facilitate selective inhibitor design. It is also possible that there are differences in biology, which may make *Plasmodium* more sensitive to inhibition of the target than the human host, particularly over the short time scale that is required for treatment of the asexual blood stage infection.

Finally, other issues considered here are TPP/TCP fit, the presence of structural information, and the availability of appropriate assay(s). For those targets where recombinant protein is difficult to produce, phenotypic assays with conditional knockdown parasites can be used to identify chemical starting points, although this may miss weaker or non-cell permeant hits.

In addition to the basic scoring system, we have decided that we need a diverse target portfolio. For example, the amino acid tRNA synthetases are robustly validated drug targets, but it is important to have a degree of diversity in any drug discovery pipeline to mitigate against the possibility of unforeseen scientific developments deprioritizing a specific target or target class. There is also an element of opportunities as well as taking risks with novel targets where there is relatively little information.

## Outcome of triaging

As a result of this target prioritization process, we classified targets as follows:

• High Priority: These are targets that have a high degree of validation and are likely to be good drug targets. There may be missing data that would be required prior to the progression of these targets into a full-scale drug discovery program. However, this is where we think resources should be prioritized, to complete validation experiments and progress them into drug discovery.

• Targets under Consideration: These are targets that look promising, but additional data are required to move them to a stage where they could be prioritized. For example, this might be missing information on TCP fit, or a lack of chemical tools.

• New and Emerging Targets: Targets requiring significant work focused on validation. It is important to highlight specific experiments required to validate/invalidate particular targets.

• Deprioritized Targets: Targets where we do not think resources are justified from a MalDA perspective. Here, there are two separate categories: (1) invalidated targets, or those unlikely to be inhibited by compounds capable of fulfilling one of the MMV TCPs, and (2) those targets where there are already several substantial development programs elsewhere. Examples include *Pf*ATP4 (where there are multiple programs ongoing)^18^ and plasmepsin X^42^ (see Table 4 for further examples).

**Table 4:** Examples of *Deprioritized Targets* for MalDA

target name (abbreviation)	Pf gene ID	reason for deprioritization
*N-*myristoyl transferase (NMT)	PF3D7_1412800	slow killer, challenges with selectivity compared to the human enzyme
P-type ATPase 4 (ATP4)	PF3D7_1211900	multiple series under investigation in the drug discovery pipeline
plasmepsin X	PF3D7_0808200	multiple series under investigation in the drug discovery pipeline
Niemann–Pick type C1-related protein (NCR1)	PF3D7_0107500	resistance risk, slow rate of kill and single-stage efficacy
dihydrofolate reductase (DHFR)	PF3D7_0417200	clinically approved inhibitor; resistant parasites widespread in the field
dihydroorotate dehydrogenase (DHODH)	PF3D7_0603300	multiple chemotypes have been developed, and resistance can arise readily
phosphatidyl inositol 4-kinase (PI4K)	PF3D7_0509800	multiple chemotypes have been developed, and resistance can arise readily

Inevitably, there is a degree of judgment and subjectivity, as information gaps will exist for most targets, and there are many aspects of *Plasmodium* biology and host–parasite interactions that are unknown. The ultimate proof of validation of any drug target is a clinical proof of concept, as shown for example with inhibitors of *P. falciparum* dihydrofolate reductase, dihydroorotate dehydrogenase, the cytochrome bc1 complex, or phosphatidylinositol 4-kinase IIIβ (PI4KIIIβ kinase).^43^

**Figure 2:**
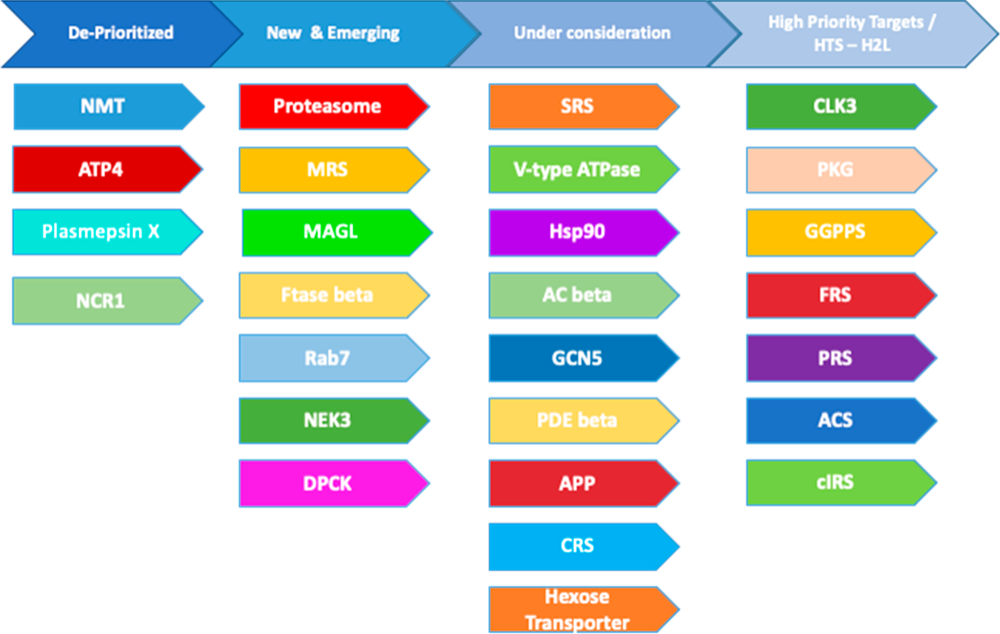
Current MalDA target portfolio

The outcome of our initial triage is shown in Figure 2. This represents the targets that we have had the opportunity to assess. There are other interesting targets that remain to be assessed by our target validation process.

Tables 5 (high-priority targets) and 6 (targets under consideration) illustrate specific details for some of the targets in Figure 2. Target prioritization is a dynamic process—prioritization of individual targets may change as key information becomes available. In Figure 2, we provide our current classification of targets. However, this will change, so we have created a Web site where this information will be updated (www.malariaDA.org). We also invite members of the malaria research community to suggest new targets, add to our assessments, and provide additional information on targets being considered by MalDA.

**Table 5:** Targets Ranked As *High Priority*

target name (abbreviation)	Pf gene ID	key questions	next steps
serine/threonine protein kinase, putative^44^ (CLK3)	PF3D7_1114700	can target be structurally enabled?	optimization of hits; more chemical starting points
cGMP-dependent protein kinase (PKG)	PF3D7_1436600	rate of kill with selective inhibitor	Rate of kill (PRR assay); optimize chemical starting points
geranylgeranyl pyrophosphate synthase (F/GGPPS)	Pf3D7_1128400	small molecule inhibitors	generate additional chemical matter
phenylalanine tRNA synthetase–alpha subunit (PheRS)	PF3D7_0109800	resistance risk	more screening/scaffold hopping to identify more start points
prolyl tRNA synthetase, putative (ProRS)	PF3D7_0925300	is selectivity versus human orthologues possible?	additional screens
acetyl CoA synthetase, putative (AcAS)	PF3D7_0627800	resistance risk; can the target be structurally enabled?	proof of concept from MMV693183 first-in-human study; establish alternate lead series from existing hits/new screens and H2L; crystal structure to guide chemistry program
isoleucine—tRNA ligase, putative (cIRS)	PF3D7_1332900	rate of kill, resistance risk	generate additional chemical matter

**Table 6:** Targets Ranked As *Under Consideration and in Assay Development and Screening Stages*

target name (abbreviation)	Pf gene ID	key questions
cytosolic seryl-tRNA synthetase (SerRS)	PF3D7_0717700.1	chemical starting points; TCP fit
V-Type H+ ATPase	includes PF3D7_0406100, PF3D7_0806800, PF3D7_1311900	generate chemical matter; understand resistance profile, druggability, and TCP fit
heat shock protein 90 (HSP90)	PF3D7_0708400	selectivity; more chemical starting points
adenylyl cyclase beta (AC beta)	PF3D7_0802600	TCP fit; more potent inhibitors; selectivity
histone acetyltransferase GCN5 (GCN5)	PF3D7_0823300	rate of kill; chemical starting points
phosphodiesterase beta (PDEβ)	PF3D7_1321500	resistance potential TCP fit; More chemical starting points; chemical tools for validation
aminopeptidase P (APP)	PF3D7_1454400	TCP fit; selectivity; chemical tools
cysteine tRNA synthetase (CysRS)	PF3D7_1015200.1	protein expressed; development of assay; selectivity
hexose transporter (HT)	PF3D7_0204700	is selectivity versus human orthologues possible? TCP fit; activity against various life cycle stages

By way of example, we will discuss three key targets. These are intended as general examples to illustrate our approach and thinking, across a range of different targets, and represent a significant focus of work from MalDA members. More information is available in the Supporting Information and on the Web site.

### Acetyl CoA Synthetase (PfAcAS) (High Priority Target)

*Pf*AcAS is responsible for the biosynthesis of acetyl coenzyme A from coenzyme A and acetate. We have recently reported the validation of this enzyme.^45,46^

#### Chemical Validation

**Figure 3:**
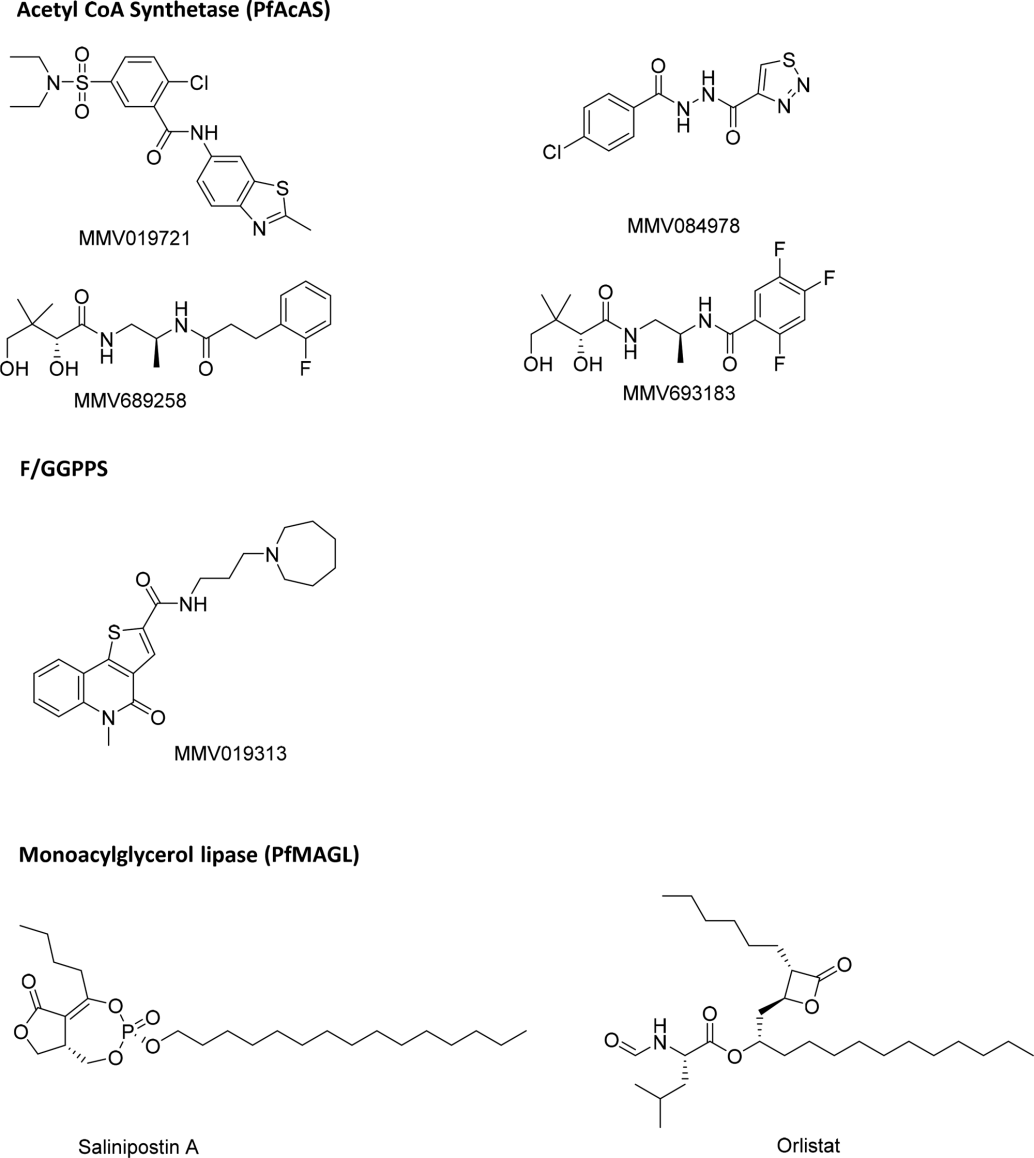
Structures of tool compounds (see text for references to each structure)

Pantothenamides such as MMV689258 and MMV693183 are converted into antimetabolites that interfere with CoA acetylation.^45^ In addition, two compounds, MMV019721 and MMV084978 (Figure 3), were found to be active in screens against both *P. falciparum* asexual blood stages (EC_50_ values of 460 nM and 370 nM, respectively) and *P. berghei* liver stages (EC_50_ values of 2100 nM and 520 nM, respectively). Generation of *in vitro* resistance to these compounds, followed by whole-genome sequencing, revealed multiple mutations in the gene encoding *Pf*AcAS. These mutations clustered around the predicted active site of the enzyme. *Pf*AcAS biochemical assays indicated that all test compounds inhibit this enzyme. Pantothenamides and MMV019721 are competitive with respect to coenzyme A, and MMV084978 displays a mix-inhibition mode with respect to acetate.

#### Genetic Validation

Several approaches have indicated the genetic essentiality of this enzyme. The enzyme was reported to be essential in *P. berghei*(34) and was predicted to have a high likelihood to be essential in a *P. falciparum* piggyBac insertion mutagenesis screen.^33^ Conditional knockdown of the enzyme led to both reduced parasite viability and increased sensitivity to pantothenamides, MMV019721, and MMV084978. In contrast, using CRISPR/Cas9 gene editing to replace the wild-type gene for *Pf*AcAS with allelic variants encoding resistance mutations led to parasites with reduced sensitivity to pantothenamides, MMV019721, and MMV084978.

#### Resistance Potential

It is possible to generate parasite cell lines *in vitro* that are resistant to MMV689258, MMV019721, and MMV084978, and studies to define the MIR values are ongoing.^35^ One of the pantothenamides shows an MIR of 10^9^. As MMV019721 and MMV084978 appear to bind to different sites on the enzyme, each may have a different resistance susceptibility.

#### Druggability

Pantothenamides, MMV019721, and MMV084978 are small molecules that fit within typical drug-like space. The pantothenamide MMV693183 meets all criteria for a preclinical candidate and is progressing toward a first-in-human study.

#### TCP fit

MMV019721 and MMV084978 are active against both asexual blood and liver stage parasites with relatively similar EC_50_ values. Pantothenamides target both asexual and sexual blood stages but have lower activity against liver stages.

#### Toxicity

Pantothenamides were very well tolerated in *in vivo* pharmacokinetic, efficacy, and exploratory toxicology studies. MMV019721 and MMV084978 are at an earlier stage in the discovery pipeline and have not been subject to a detailed assessment of toxicity. However, there is a high degree of selectivity at both the enzyme level (compared to human acetyl CoA synthetase) and cellular levels (compared to HepG2 cells).

#### Novelty

This is a novel target for malaria. There are reports of inhibitors of fungal AcAS enzymes.^47^

#### Assay Readiness

Both *Pf*AcAS and *Hs*AcAs have been recombinantly expressed and assays developed for high-throughput screening.

#### Structural Information

There is no structural information for *Pf*AcAS, which to date is proving challenging to crystallize, in our hands.

As a result of all of these studies, there is strong evidence that *Pf*AcAS is a good drug target and is druggable. The pantothenamide MMV693183 is currently in preclinical development. The identification of alternate series capable of inhibiting *Pf*AcAS would be greatly facilitated by structural information and information regarding inhibitor binding. More work is also required to assess the resistance risk across inhibitor classes. This target has now progressed into drug discovery through MalDA-supported efforts.

### Bifunctional Farnesyl/Geranylgeranyl Pyrophosphate Synthase (F/GGPPS; High Priority Target)

Bifunctional farnesyl/geranylgeranyl pyrophosphate synthase is a key enzyme in isoprenoid biosynthesis that synthesizes C15 and C20 prenyl chains. Prenyl chains are the substrates of a number of prenyltranferases resulting in isoprenoid products essential for parasite survival.

#### Chemical Validation.^48^

MMV019313 (Figure 3) inhibits F/GGPPS activity in enzymatic assays. Generation of *in vitro* resistance to this compound, followed by whole-genome sequencing, revealed mutations in F/GGPPS (S228T). Overexpression of F/GGPPS in parasites was sufficient to confer resistance to MMV019313. Overexpression of the F/GGPPS(S228T)-GFP variant, even at moderate levels, resulted in an 18-fold increase in the EC_50_ value of MMV019313.

#### Genetic Validation

The essentiality of this enzyme has been demonstrated via several different approaches. The *P. berghei* genome-wide screen showed it to be essential.^34^ The *P. falciparum* PiggyBac insertion mutagenesis screen shows that the enzyme is nonmutable in its coding sequence.^33^ Conditional knockdown studies confirm essentiality *in vitro*. S288T allelic replacement parasite lines recapitulate the resistance phenotype of drug-selected lines. Further studies are required to ascertain whether these changes represent indirect resistance mechanisms as opposed to being mutations in drug targets

#### Resistance Potential

Parasites resistant to MMV019313 acquired a S228T mutation in this gene. A 10-fold shift in susceptibility was observed compared to the wild type. The MIR was determined to be 10^8^.

#### Druggability

There is current confidence that drug-like compounds can be identified. MMV019313 is a small molecule within the drug-like space even if it needs optimization to become a drug, suggesting that small molecule inhibitors can be identified.

#### TCP fit

MMV019313 shows asexual blood stage activity (EC_50_ 270 nM) and complies with TCP-1. The compound has not yet been tested for gametocyte activity. It is not active against liver stages.

#### Toxicity

Human orthologues exist, and assays are available. MMV019313 inhibits the enzymatic activity of *Pf*F/GGPPS but not human FPPS or GGPPS.

#### Novelty

*Pf*F/GGPPS is a validated target for malaria. Current drugs for this target in other disease areas show poor bioavailability and selectivity. The new tool compound is a small molecule that is selective and has a distinct mode of inhibition compared to current drugs.

#### Assay Readiness

Various assay options have been reported, including pyrophosphate production^48^ and radiolabeled methods.^49^

#### Structural Information

A crystal structure is available for *Pv*F/GGPPS, which allows for the generation of structural models for other *Plasmodium* homologues.

The discovery of a new small molecule binding to a novel site, with superior druggability to that of the previously established inhibitor binding site, makes this target very attractive.

### Monoacylglycerol Lipase PfMAGL (New and Emerging)

Human monoacylglycerol lipase (MAGL) catalyzes the hydrolysis of a variety of monoglycerides into fatty acids and glycerol. In *P. falciparum*, this enzyme has been reported to play a role in processing these monoglycerides, including palmitoyl and oleoyl glycerols.^50^

#### Chemical Validation

The natural product, salinipostin A,^51^ and the lipid metabolism inhibitor, orlistat (Figure 3),^50^ were shown to be potent against *P. falciparum* asexual blood stage W2 parasites with EC_50_ values of 50 nM and 280 nM, respectively. Potent inhibition of recombinant *Pf*MAGL using hits from a small library of triazole–urea inhibitors strongly correlated with *in vitro* antiplasmodial activity against W2 parasites.

#### Genetic Validation

Genetic essentiality has been shown in the *P. falciparum* piggyBac screen.^33^

#### Resistance Potential

*In vitro* resistance selection experiments with salinipostin A using a high parasite inoculum (10^9^) in a genetically engineered Dd2 parasite line with an increased mutation rate (Dd2_Polδ) yielded parasites that showed 2- to 9-fold reductions in sensitivity. However, no mutations were observed in *Pf*MAGL in this experiment. Instead, all resistant parasites cloned had SNPs in the “protein of relevant evolutionary and lymphoid interest” (PRELI) domain-containing protein of unknown function (PF3D7_1324400) with five of six clones harboring A20V, P102L, and T145I mutations in the coding sequence, while the sixth clone had a point mutation in intron 1 that has an unknown impact on expression levels. Mutations at S725Y and E507K in the V-type H^+^-translocating pyrophosphatase (PF3D7_1235200) were also observed in three out of six clones.

#### Druggability

Salinipostin A is a natural product, while orlistat is prescribed for obesity. The potent activity of these compounds against *P. falciparum* proliferation and recombinant *Pf*MAGL highlights the feasibility of exploring their respective scaffolds for structure–activity relationship studies to design specific small molecule inhibitors of *Pf*MAGL. However, new series that are more attractive from a medicinal chemistry perspective would increase the druggability confidence.

#### TCP Fit

Salinipostin A and orlistat are active against asexual blood stages at nanomolar EC_50_ values. However, the activity of these compounds against liver stage parasites and gametocytes has yet to be investigated.

#### Toxicity

Salinipostin A has a selectivity index >1000 for *P. falciparum* asexual blood stage parasites compared to a variety of mammalian cell lines including human foreskin fibroblasts (HFF), HEK293T (human kidney), U2OS (human osteosarcoma), and AsPC-1 (human pancreatic adenocarcinoma).^51^

#### Novelty

This is a novel target for malaria.

#### Assay Readiness

There already exist platforms to study this enzyme, including a fluorogenic substrate assay to assess the characteristics of the recombinant *Pf*MAGL with various fluorogenic lipid ester substrates bearing different chain lengths. Parasite lines overexpressing *Pf*MAGL from an exogenously transfected plasmid have been developed. In addition, there is a competition assay for active site labeling of *Pf*MAGL and human MAGL with the broad-spectrum serine hydrolase ABP fluorophosphonate-rhodamine (FP-Rho) to screen compounds for their ability to compete for the active sites of the two enzymes and identify highly potent and selective hits.

#### Structural Information

The crystal structure of the human MAGL is available (PDB: 3jw8) and a predicted *Pf*MAGL homology model templated on this structure has been published.^50^

This target shows promise as an antimalarial target, based on the good correlation between enzyme and parasite inhibition and the low propensity of resistance for these inhibitors. The availability of assays will facilitate screens to identify new potent and selective hits. Further experiments are required to complete the chemical and genetic validation and to assess the TCP fit for this target.

## Future Perspectives

### Phenotypic Screening

In the past 20 years, drug discovery against malaria has largely focused on phenotypic screening approaches, due to the relative lack of robustly validated targets. Many of the readily available compounds in lead-like and drug-like space have already been screened against *P. falciparum*, leading to a lack of novel compound libraries to screen, particularly those addressing novel areas of chemical space. Therefore, an increased effort focusing on target-based approaches is merited. In the past 10 years, significant progress has been made in identifying and validating potential drug targets for malaria. Information about antimalarial drug targets is being captured in a Web site by the International Union of Basic and Clinical Pharmacology (https://www.guidetomalariapharmacology.org/malaria/).

It is important that the most appropriate drug targets are carefully selected for progression into drug discovery. Despite the success in identifying clinical candidates for malaria, we must be mindful of the high attrition rate in clinical development, as is seen for all drug development programs. For infectious diseases, the typical success rate from entry to phase 1 clinical studies to compound registration is 19–25%.^52,53^ Success in drug discovery and subsequent clinical development is highly dependent upon both the selection of the molecular target and the properties of the compounds progressed.

The properties of a particular molecule will be at least in part dependent on the druggability of the target. Key challenges in molecular designs for antimalarial drug discovery include compounds with a long half-life, suitable for single-dose oral treatment and chemoprotection; oral bioavailability; stability for storage at room temperature in tropical conditions for extended periods; a low cost of goods; and good safety profiles.

Resistance is a key issue, particularly for asexual blood stage infections. It is not totally clear whether resistance is solely a function of the target or the compound or both. Some targets are known to be particularly susceptible to mutations (for example, dihydroorotate dehydrogenase). For targets with several different drug binding pockets (e.g., tRNA synthetases; due to multiple substrates), each may have a different risk of mutation.

As well as identifying the most promising molecular targets, we also need to identify for each target the most appropriate hit and drug discovery strategies to derive therapies with desired profiles. For example, fragment-based drug discovery approaches can be useful to optimize the physicochemical properties of molecules as they are developed.

### How Do We Identify Additional Drug Targets in Malaria?

First, there are still many phenotypically identified compounds for which the mode of action remains to be determined. Some of these may be acting on more than one target or acting on nonprotein targets. Compounds that demonstrate polypharmacology may be desirable to reduce the resistance risk; however, the design of such molecules is in its early stages.

**Figure 4:**
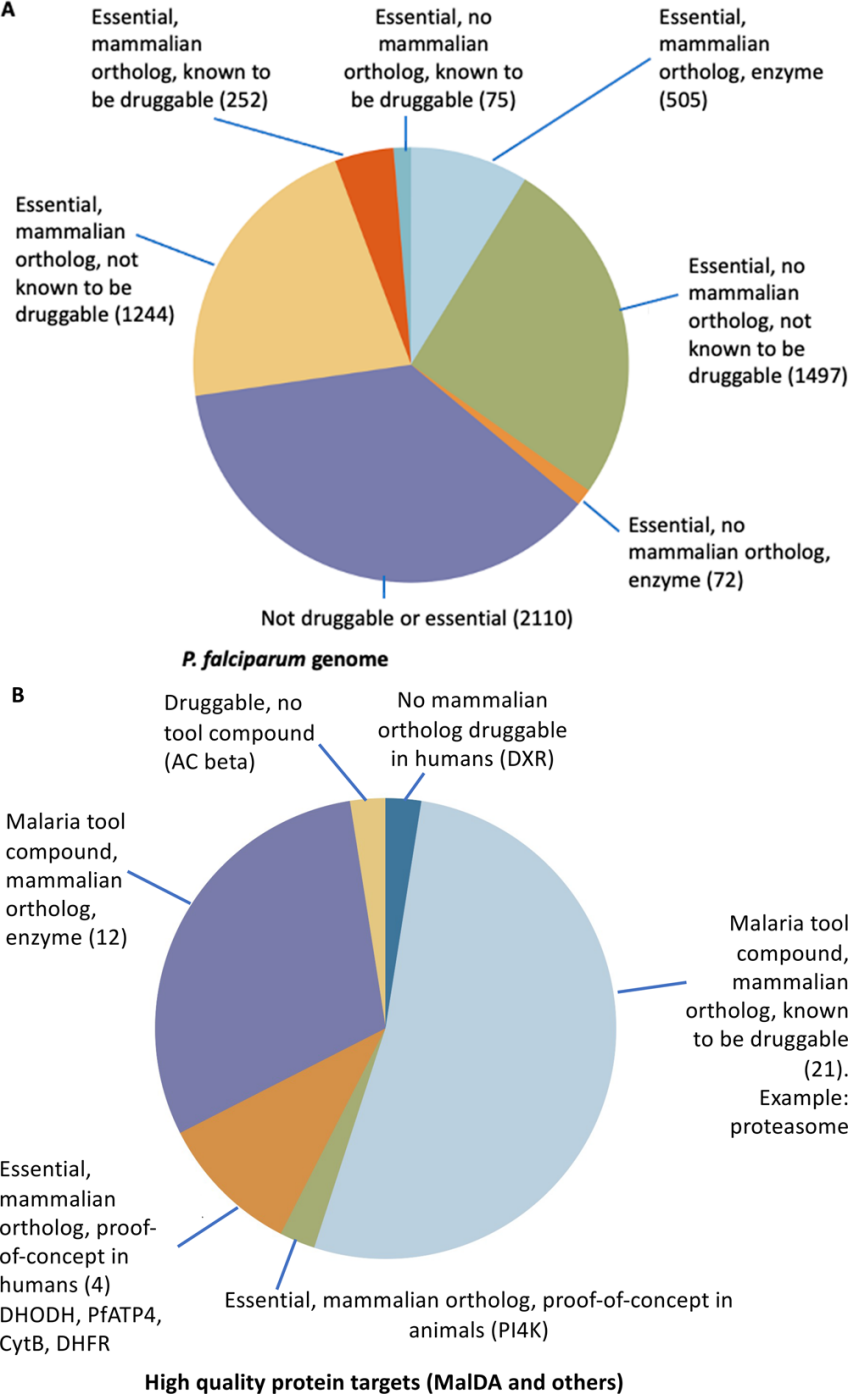
(A) Analysis of the *P. falciparum* genome categorizing targets according to their predicted essentiality, druggability, and the presence of mammalian orthologs. The number of proteins in each category is shown in parentheses. (B) Analysis of high value targets, according to tool compounds, the presence of mammalian orthologs, and proof of concept in humans. The number of proteins in each category is shown in parentheses. Where there is only one protein in a category, it is stated explicitly. Most MalDA targets fall into the light blue or violet regions.

Second, we are investigating a “big-data” approach to identify potential new targets. As an initial triage, we have analyzed the *P. falciparum* genome and cross-referenced this against a number of different databases (Figure 4A), to identify targets that it might be valuable to investigate. The workflow is as follows:

•Essentiality. To determine if the target is essential in *Plasmodium*, we have used the data obtained through saturation mutagenesis^33^ or functional analysis.^34^

•Druggability. We used the recently published method of Wang and coauthors to assess ligand binding ability.^54,55^ We extracted the 236 InterPro domains listed as ligandable and searched PlasmoDB for proteins with these domains.

•Enzymes. We also included proteins annotated with an EC number or predicted to catalyze a reaction because many are known to be good drug targets. Many enzymes were also considered ligandable using the method above (e.g., kinases).

•Mammalian Orthologue. We examined whether a human orthologue exists using the ortholog search function within PlasmoDB.^56^ The lack of a mammalian orthologue has traditionally been considered an attractive feature for anti-infective drug targets. However, it is important that the presence of a human orthologue is not used as a reason for deprioritizing a target: (1) Where there is a human orthologue, it is very often possible to obtain high levels of selectivity (for example, against *P. falciparum* dihydrofolate reductase). (2) Sometimes there are very different levels of “vulnerability” between a pathogen enzyme and its orthologues. (3) *Plasmodium* targets for which there is not a human orthologue are not always attractive targets. For example, many early, postgenomic drug discovery programs were focused on members of the nonmevalonate pathway of isoprenoid biosynthesis,^57^ or other proteins targeted to the apicoplast (including bacterial-type proteins involved in protein biosynthesis), a parasite-specific organelle involved in the production of isoprenoids.^58^ While it may be possible to find highly selective inhibitors for such targets, the rate of kill may be slower, as was the case for fosmidomycin (that targets DOXP reductoisomerase), tetracycline, or azithromycin.^59^ Another caveat is that some of these druggable parasite-specific targets, such as enoyl-ACP reductase (FabI, PF3D7_0615100), may only be important for the liver or sporozoite stage.^60,61^

This analysis suggests that there are about 300–700 potential drug targets in *Plasmodium*. These will have different profiles, and inhibitors of these may fulfill different TCPs.

We then did an analysis of the malaria protein targets that have extensive validation, including those with proof-of-concept data in humans (Figure 4B). These include the targets that MalDA has assessed as being high value (Figure 2), along with targets known from current drugs and targets from compounds currently in clinical development, including PI4K, eEF2, and DHODH.^43^

In addition to the annotated *Plasmodium* genes, there are many genes that have not been annotated, the so-called hypothetical proteins. There may well be attractive targets, which could be very different structurally and functionally from human proteins, potentially allowing for selective inhibitors. It is also important to consider that the definition of druggability, for this exercise, is dependent on the existence of ligand-bound protein structures in the Protein Data Bank (https://www.rcsb.org/). Proteins that are more difficult to structurally characterize, such as membrane proteins, may have been overlooked. Paradoxically, the more different a *Plasmodium* protein is from its mammalian ortholog, the more likely it is to not be recognized as druggable. Another important consideration is that there may be nonprotein targets, including rRNAs. Compounds that target the bacterial-type protein biosynthesis machinery, including azithromycin, clindamycin, and tetracycline, have antimalarial activity, although many have a slow rate of kill.

While the focus of this exercise has been to prioritize potential targets for drug discovery, this process will have considerable value in further dissecting the fundamental biology of these parasites. Understanding the biological context of a target is key to understanding how a potential drug target may be exploited in the clinic. Further, since all antimalarials are now given as combination therapies, understanding the background biology is important in selecting combinations. Even for targets that are not deemed suitable from a drug discovery context, this exercise can still provide valuable biological insights into these important pathogens.

One aim of this publication is to stimulate discovery and validation of potential new drug targets for *Plasmodium*. We hope that it will facilitate efforts in the broader malaria community writ large to continue to contribute valuable insights to support the antimalarial drug development effort. We welcome comments from the community on these and other targets. To that end, we invite comments on our Web site (www.malariaDA.org), which will then be used to annotate a list of potential targets, to be displayed on the Web site.

## Supporting Information Available

Assessments of other molecular targets (PDF)
