# Supplementary material for: Prioritization of Molecular Targets for Antimalarial Drug Discovery
Source: ACS Infect Dis. 2021 Sep 15;7(10):2764–76. doi: 10.1021/acsinfecdis.1c00322 (PMC8608365; doi:10.1021/acsinfecdis.1c00322)
Supplement: Supplementary file 1 — id1c00322_si_001.pdf [file id1c00322_si_001.pdf]

SUPPORTING INFORMATION

Prioritisation of Molecular Targets for Antimalarial Drug Discovery

Barbara Forte,[a,#] Sabine Ottilie,[b,#] Andrew Plater,[a,#] Brice Campo,[c] Koen Dechering,[d] Francisco Javier Gamo,[e] Daniel E. Goldberg,[f] Eva S. Istvan,[f] Marcus Lee,[g] Amanda K. Lukens,[h,i] Case W. McNamara,[j] Jacquin C. Niles,[k] John Okombo,[l] Charisse Flerida A. Pasaje,[m] Miles G. Siegel,[n] Dyann Wirth,[h,i] Susan Wyllie,[a] David A. Fidock,[l,n] Beatriz Baragaña,[a,*] Elizabeth A. Winzeler,[b,*] Ian H. Gilbert[a,*]

Affiliation

a. Wellcome Centre for Anti-Infectives Research, Division of Biological Chemistry and Drug Discovery, University of Dundee, Dundee, DD1 5EH, UK
b. Department of Pediatrics, School of Medicine, University of California, San Diego, La Jolla, California 92093, USA
c. Medicines for Malaria Venture, 1215 Geneva, Switzerland.
d. TropIQ Health Sciences, 6534 AT, Nijmegen, The Netherlands
e. Global Health, GSK. 28760-Tres Cantos, Madrid, Spain
f. Division of Infectious Diseases, Department of Medicine and Department of Molecular Microbiology, Washington University School of Medicine, St. Louis, Missouri 63110, USA.
g. Wellcome Sanger Institute, Wellcome Genome Campus, Hinxton, CB10 1SA, UK
h. Infectious Disease and Microbiome Program, Broad Institute, Cambridge, MA 02142, USA
i. Department of Immunology and Infectious Diseases, Harvard T.H. Chan School of Public Health, Boston, MA 02115, USA
j. Calibr, a division of The Scripps Research Institute, 11119 North Torrey Pines Road, La Jolla, CA 92037, USA
k. Department of Biological Engineering, Massachusetts Institute of Technology (MIT), Cambridge MA 02139-4307, USA
l. Department of Microbiology and Immunology, Columbia University Irving Medical Center, New York, NY 10032, USA
m. Lgenia, Inc., Fortville, IN 46040, USA
n. Division of Infectious Diseases, Department of Medicine, Columbia University Irving Medical Center, New York, NY 10032, USA

# Contributed equally to this work
* Authors for correspondence: b.baragana@dundee.ac.uk; ewinzeler@ucsd.edu; i.h.gilbert@dundee.ac.uk

Example Assessments of Additional Drug Discovery Targets

Cyclin Like Kinase 3 (CLK3) (High Priority Target) 3
Cyclic GMP-dependent protein kinase (PKG) (High Priority Target) 4
Cytoplasmic Prolyl tRNA Synthetase (ProRS) (High Priority Target) 6
Cytoplasmic Phenylalanine tRNA Synthetase (PheRS) (High Priority Target) 7
HSP90 (Target Under Consideration) 8
Farnesyltransferase (Target Under Consideration) 9
Hexose Transporter (PfHT) (Target Under Consideration) 10
Niemann-Pick NCR1 (Target Deprioritised) 11

CLK3

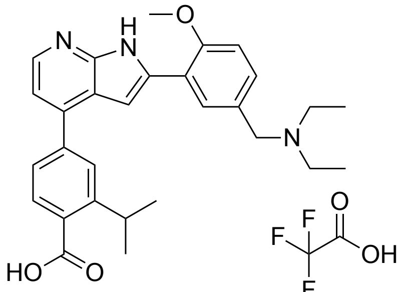

TCMDC-135051

PKG

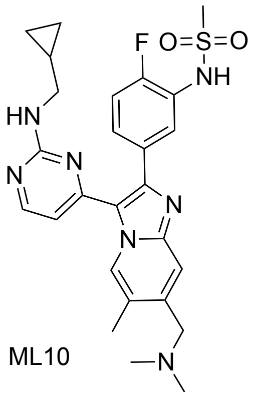

ML10

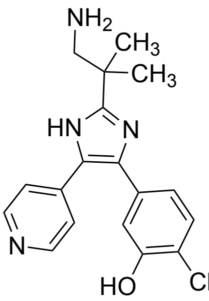

MMV030084

ProRS

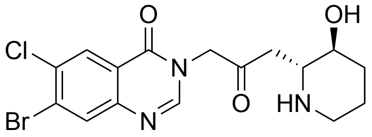

Halofuginone

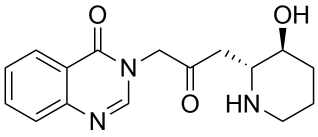

Febrifugine

PheRS

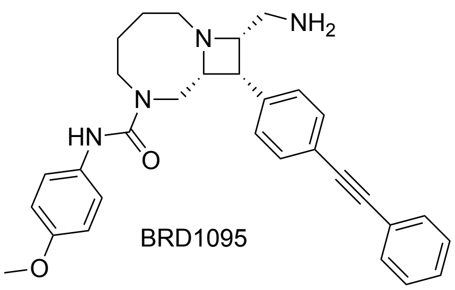

BRD1095

NCR1

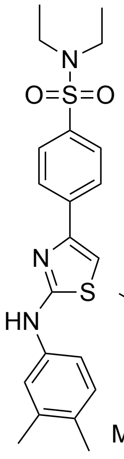

MMV009108

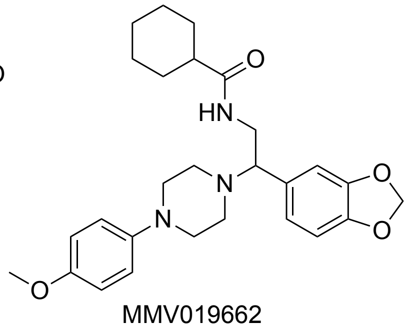

MMV019662

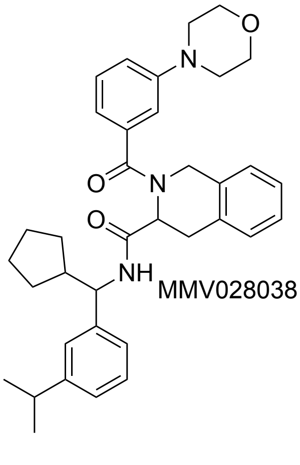

MMV028038

Farnesyltransferase

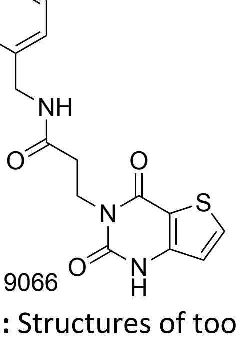

MMV019066

HSP90

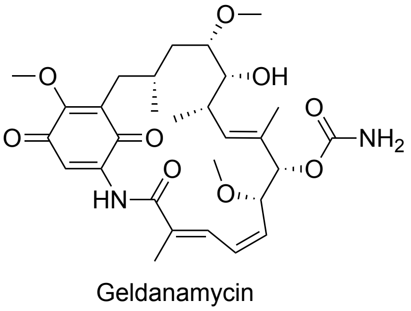

Geldanamycin

Figure S1: Structures of tool compounds

Cyclin Like Kinase 3 (CLK3) (High Priority Target)

PfCLK3 is one of the four cyclin-dependent like protein kinases in *Plasmodium*. *Pf*CLK3 with other members of the *Pf*CLK family plays a key role in the processing of parasite RNA.

- **Chemical Validation**.[1] TCMDC-135051 (Figure **S1**) is active *in vitro* against recombinant *P. falciparum*, *P. berghei* and *P. vivax* CLK3 with EC_{50} values of 110 nM, 33 nM and 13 nM respectively. TCMDC-135051 is also active against *P. falciparum* asexual blood stages (EC_{50} 110 nM), the *P. berghei* liver stages (EC_{50} 400 nM) and *P. falciparum* gametocytes (stage II) (EC_{50} 800 nM). Generation of *in vitro* resistance to this compound, followed by whole-genome sequencing revealed mutations in *Pf*CLK3. Twice-daily intraperitoneal dosing of TCMDC-135051 into mice infected with *P. berghei* resulted in a dose-related reduction in parasitaemia over a 5-day infection period, where the maximal dose (50 mg/kg) resulted in near-complete clearance of parasites from peripheral blood.
- **Genetic Validation**.[1] The essentiality of this enzyme has been demonstrated by a variety of different experiments. Conditional knockdown shows essentiality *in vitro*. PiggyBac insertion mutagenesis screen shows the enzyme has a medium likelihood to be essential. Generating mutant parasite lines by allelic replacement with the resistance mutation (G449P), recapitulates the resistance phenotype observed *in vitro*. Enzymatic assays show a shift in sensitivity to TCMDC-135051.
- **Resistance Potential**. Parasites resistant to TCMDC-135051 were generated and showed mutations outside of the CLK3 kinase domain (P196R and H259P). EC_{50} fold shift of resistant mutants are between 5 (for the parasite line containing the P196R mutation) and 13-fold (with the H259P variant).[1] The MIR was estimated at 10^9. G449P mutant parasites demonstrate no apparent fitness defect compared to wild-type in asexual blood stage growth assays.
- **Druggability**. TCMDC-135051 is a small drug-like molecule, even if it requires optimisation to become a candidate. Furthermore, protein kinases are known to be druggable.
- **TCP fit**. TCMDC-135051 demonstrates multistage activity against asexual blood and liver stages and transmission-blocking activity.
- **Toxicity**. TCMDC-135051 demonstrates very good selectivity against *Pf*CLK3 compared to the human orthologue PRPF4B and the closely-related human kinase CLK2.[1]
- **Novelty**. *Pf*CLK3 is a novel target for *P. falciparum*.
- **Assay Readiness**. Recombinant expression of wild-type PfCKL3 has been demonstrated in *E. coli* along with a high throughput screening assay on the LANCE® platform.[1]
- **Structural Information**. A crystal structure is not available, but a homology model is available.

Collectively, these data show that targeting *Pf*CLK3 is a novel strategy with a high potential to find a new antimalarial drug with multistage activity.

Cyclic GMP-dependent protein kinase (PKG) (High Priority Target)

PKG is a serine/threonine protein kinase that acts as a key regulator of cGMP signaling. In *P. falciparum*, this kinase plays an essential role in multiple stages of the parasite life cycle, including egress and invasion as well as in gametogenesis,[2,3] all of which are critical for parasite proliferation and transmission.

- **Chemical validation**: Chemical inhibition of *Pf*PKG was first demonstrated using ML10 (Figure S1), the lead compound from an imidazopyridine series of PKG inhibitors, which displayed picomolar activity against recombinant *Pf*PKG and *in vivo* parasite clearance in a humanized mouse model of *P. falciparum* infection.[4] More recently, MMV030084 (Figure S1), a trisubstituted imidazole with activity against multiple stages of the parasites, was identified as a PKG inhibitor by deploying a multi-omics approach to deconvolute its mode of action.[5] Biochemical assays with this compound demonstrated sub-nanomolar potency against *Pf*PKG. Using *P. falciparum* transgenic lines in which the small gatekeeper T618 residue was substituted with a bulkier glutamine through allelic replacement at the endogenous PKG locus, parasites expressing the PKG 618Q allele were reported to exhibit multi-fold loss in sensitivity to PKG inhibitors compared with parental wild-type parasites.[3]
- **Genetic validation**: PKG was shown to be essential by several genetic methods, including the genome-wide piggyBac screen,[6] and by conditional knockdowns (cKD) using CRSIPR/Cas9 to introduce aTc-dependent downregulation of PKG expression. Under non-permissive conditions parasite growth was 97% inhibited compared with controls, and >4-fold sensitization to MMV030084 was observed in PKG cKD lines compared to unmodified controls.[5]
- **Resistance Potential**: Although selection experiments with Dd2-B2 parasites and MMV030084 and the imidazopyridine ML10 yielded a resistant line with a ~3-fold shift in IC_{50} compared to the parental line,[5] they did not give any mutations in PKG. However, whole-genome sequencing identified a T1268R mutation in tyrosine kinase-like protein 3 (TKL3, PF3D7_1349300), which was validated using CRISPR/Cas9 gene editing as causal for low-level resistance to PKG inhibitors. This is suggestive that either PKG is a target with a low propensity for resistance or that MMV030084 inhibits multiple targets, possibly a diverse set of protein kinases.
- **Druggability**: Protein kinases are a known druggable target class, with many examples of clinically used drugs acting against human targets.[7] The ATP binding site present in all members of the family is structurally well understood, and several distinct binding modes have been determined.[8,9] The druggability of *Pf*PKG using small molecule ATP-competitive inhibitors has been shown through the potency of ML10 and MMV030084. A recent target-based biochemical screen of the GlaxoSmithKline (GSK) Full Diversity Collection against recombinant *Pf*PKG also identified potent trisubstituted thiazoles, with the frontrunner compound exhibiting enzyme and whole cell inhibition with nanomolar potency.
- **TCP fit**: MMV030084 showed an EC_{50} of 199 nM against *P. berghei* liver-stage parasites and an EC_{50} < 150 nM against *P. falciparum* asexual blood stage parasites. In addition, MMV030084 inhibited male gametogenesis *in vitro* at an EC_{50} of 141 nM, highlighting its utility as an antimalarial candidate with prophylactic, anti-asexual blood stage and transmission-blocking properties.
- **Toxicity/selectivity**: The large complement of the human kinome presents challenges with respect to obtaining selectivity at the ATP binding site. However, key structural differences, particularly in the size of the gatekeeper residue, may be exploited[10] to develop selective compounds. The structure of *Pf*PKG has also been reported.[11] In addition, there is a wealth of information on human kinases due to interest in their role in human diseases.[12-14] The presence of a smaller residue at the gatekeeper position in *Pf*PKG avails a unique small

hydrophobic pocket that can be occupied by a small molecule ATP-competitive inhibitor thereby conferring selectivity. Targeting the ATP site of PKG is one strategy. The regulation of PKG by cyclic GMP also offers an alternative approach to designing ATP-competitive inhibitors. Allosteric modulation via the cGMP binding site has been demonstrated and potentially offers superior selectivity,[15] but potent antimalarials acting via this mechanism have not yet been reported. In addition, *Plasmodium* and human PKG are reported to differ in the activation mechanism and regulatory site specificity.[16] MMV030084 showed potent inhibition of recombinant PKG (IC_{50} 0.4 nM) but exhibited an IC_{50} of 41.5 µM against HepG2 cells, providing evidence of low toxicity and antiplasmodial selectivity.

- **Novelty**: PKG is a novel antimalarial target as none of the current antimalarial drugs in clinical use inhibit this enzyme. Moreover, none of the standard antimalarials or any experimental compounds in advanced stage development contain similar substructures to the tool compounds ML10 and MMV030084, thus minimizing the likelihood of cross-resistance.
- **Assay Readiness**: Multiple technologies exist for assaying protein kinases, usually involving a small peptide phospho-acceptor, with ATP as the phospho-donor substrate. While historically the radiolabelled separation assay is the gold standard, other platforms are more amenable to high-throughput screening, for example HTRF®, ALPHAScreen™, or ATP consumption assays such as Promega’s ATP-Glo™. The increasing use of mass spectrometry for assay read-outs has also encompassed protein kinases.[17] *Pf*PKG has been expressed and purified in amounts sufficient to support HTS campaigns. Several cell lines have also been developed and optimized for studying this enzyme, including conditional knockdown lines and CRISPR/Cas9 engineered lines expressing mutant TKL3 alleles that confer resistance to MMV030084.
- **Structural Information**. The cGMP-free crystal structures of PKG from *P. falciparum* (PDB: 5DYK) and *P. vivax* (PDB: 5DYL) are available - revealing how key structural components are arranged in the enzyme’s inactive state.[4,11] Molecular modelling showed docking of MMV030084 and its analogue into the ATP-binding pocket of PKG.

A recent review of kinases as malaria targets provides insight into the progress that has been made for several *Plasmodium* kinases, including PKG.[18] Altogether, these findings justify the attractiveness of *Pf*PKG as an antimalarial target and highlight the potential of ML10 and MMV030084 as good starting points for enhanced medicinal chemistry efforts to support lead development.

Cytoplasmic Prolyl tRNA Synthetase (ProRS) (High Priority Target)

ProRS is a member of the tRNA synthetase family, many of which are attractive targets for human diseases.[19] These proteins are responsible for charging amino acids to their cognate tRNA molecules for protein synthesis.

- **Chemical Validation**. ProRS has been chemically validated by studies with halofuginone (Figure S1) an analogue of febrifugine (Figure S1) that has long been known to possess antimalarial activity. However, this molecule also exhibits potent human ProRS activity, resulting in toxicity.[20]
- **Genetic Validation**. ProRS was shown to be essential by the piggyBac insertion mutagenesis screen.[6]
- **Resistance Potential**. Resistance mechanisms have been elucidated for ProRS; notably the short-term exposure of asexual blood stage parasites to halofuginone results in a large increase in the intracellular proline concentration,[21] thereby reducing the compound's efficacy. As halofuginone binds to the proline-binding site in an ATP-dependent manner,[22] this mechanism of resistance suggests that inhibition through binding to the proline site is not suitable.
- **Druggability**. Drug-like molecules exist for ProRS, and for other tRNA synthetase enzymes across various species.[23,24] There are also approved antifungals that inhibit aaRS enzymes.[25]
- **TCP fit**. This target has the potential to fulfil TCP1 or TCP4.
- **Toxicity**. Halofuginone exhibits limited selectivity over the human enzyme. Selectivity is a key criterion for advancing any inhibitor for this target.
- **Novelty**. ProRS is not the target of known antimalarial drugs. aaRS are known targets in other microbes and this class has been proposed as a target in malaria.
- **Assay Readiness**. *Pf*ProRS has been expressed recombinantly to facilitate high-throughput screening and crystallography.[26] Assay options for high-throughput screens include ATP depletion assays and phosphate generation assays.[27,26]
- **Structural Information**. The crystal structure of *Pf*ProRS has been solved, along with the human orthologue, allowing a detailed structural comparison. The enzymes show a high degree of identity, and the residues involved in binding halofuginone are identical between the human and *Plasmodium* forms, making it unlikely that selective analogues of this molecule could be developed. However, there are differences in the active site that could potentially be exploited, and allosteric inhibitors have been identified for ProRS that demonstrate much greater selectivity for *Pf*ProRS over HsProRS.[25]

Inhibition of this target is likely to be lethal for the parasite. The key issue is to improve selectivity against the human orthologue. Inhibitors that bind to the proline-binding site are unlikely to be efficacious due to the significant accumulation of proline that results from inhibition of ProRS. Accumulated proline will then compete with any inhibitor for binding at this site.

Cytoplasmic Phenylalanine tRNA Synthetase (PheRS) (High Priority Target)

- **Chemical Validation**. *Pf*PheRS has been validated through compounds from the Broad Institute "Diversity Oriented Synthesis" library.[28] The target of the compounds was identified by resistance generation followed by whole-genome sequencing.
- **Genetic Validation**. This target has been genetically validated via a variety of approaches. It is annotated as essential in the Plasmogem database and was found to be essential in the piggyBac screen.[6] Conditional knockdown experiments also indicate that the enzyme is essential (unpublished data). This is not perhaps surprising, given the essential role of tRNA synthetases in protein synthesis and the fact that all others investigated appear essential.
- **Resistance Potential**. In an assay to measure resistance generation, this was a low risk for resistance.[28,29]
- **Druggability**. The compounds produced by the Broad Institute, which inhibit *Pf*PheRS are drug-like.
- **TCP fit**. These compounds also are active against liver schizonts and stage IV-V gametocytes, although there is some reduction in potency, particularly for the gametocyte assays. Compounds were active in mouse chemoprotection and transmission assays. These data support the potential for multi-stage activity.[28]
- **Toxicity**. BRD1095 and other analogues showed a high degree of selectivity at both enzyme and cellular levels, suggesting that toxicity due to inhibition of the host orthologue can be circumvented.[30]
- **Novelty**. There are no clinical candidates against this target. To our knowledge the most advanced project targeting this enzyme is that from the Broad Institute, which is now being conducted in collaboration with Eisai (Japan).
- **Assay Readiness**. Assays have been developed for PheRS, including phosphate production and ATP depletion assays.[30,31]
- **Structural Information**. A crystal structure has just been published, which indicates that the Broad compound series binds across both the *L*-phenylalanine binding pocket and an auxiliary site, adjacent to the adenine and *L*-phenylalanine binding sites.[30] The structural information rationalises the observed selectivity for the *Plasmodium* enzyme, in that the auxiliary pocket is very different in the human enzyme. This is a druggable target with a high degree of validation.

HSP90 (Target Under Consideration)

Heat shock protein 90 (HSP90) is an ATP-dependent molecular chaperone. HSP90 supports the folding and refolding of proteins to maintain cellular protein integrity, notably for several transcription factors and kinases.[32] There are different isoforms of HSP90 in both the human and *Plasmodium* genomes[33,34] with different cellular localisations and specific client proteins. Some isoforms are induced in response to stress, while others are constitutively expressed. The isoform most studied is the inducible *Pf*HSP90, which is analogous to the human cytosolic HSP90α.

- **Chemical Validation**. Several small molecule inhibitors, including the antibiotic geldanamycin (Figure S1), have been shown to inhibit *Pf*HSP90 *in vitro* with nanomolar potency, and to disrupt blood and liver stage parasite growth with sub-micromolar potency.[35]
- **Genetic Validation**. The genome-wide studies performed in *Plasmodium* species suggest that disruption of this gene results in impaired parasite growth.
- **Resistance Potential**. Preliminary studies place this MIR at > 8.
- **Druggability**. Molecules that interact with the ATP binding site are known, and many of these have been considered in clinical trials for anti-tumour activity, suggesting that drug-like compounds could be developed for this target.[35,36] HSP90 is generally known to be druggable.
- **TCP fit**. Geldanamycin has been shown to inhibit *P. falciparum* asexual blood stage growth with an EC_{50} of 200 nM and cause a transition block at the ring stage.[37] Inhibition of liver stages has also been demonstrated.[35]
- **Toxicity**. A number of HSP90 inhibitors have been assessed in human clinical trials for cancer but have so far failed as monotherapies due to dose-limiting toxicities.[36] The human and *Plasmodium* forms of HSP90 share a high degree of identity and are inhibited to similar degrees by geldanamycin. Other compounds known to bind to human HSP90 are also shown to interact with *Pf*HSP90. It has also been reported that the *h*ERG channel is dependent on HSP90α for maturation and trafficking.[38] However, compounds that show selectivity between the different human HSP90 isoforms have been identified, and non-ATP site binders have also been developed, indicating that selectivity might be possible.[35,36,39]
- **Novelty**. Inhibitors of HSP90 have been shown to act synergistically with other compounds,[35,40,41] suggesting a potential role for inhibitors of this target as adjunct drugs in restoring sensitivity to other drugs where resistance is already a concern.[34] Currently, there are no inhibitors of HSP90 approved for use.
- **Assay Readiness**. Recombinant *Pf*HSP90 protein has been generated using standard expression systems, and assays have been established based on fluorescence polarisation in a competitive binding platform.[35] The ATPase activity of HSP90 can also be assessed, for example by measuring the release of inorganic phosphate.[42] Luciferase-based protein denaturation assays are also known[43] and have been translated to cellular applications.[44] Various other assays have been described and reviewed.[45]
- **Structural Information**. The structure of the N-terminal ATP binding domain have been solved for human and *Plasmodium* HSP90.[39,46]

The studies conducted to date suggest *Pf*HP90 is an interesting target and that compounds targeting this chaperone may act synergistically with other drugs to reduce resistance propensity. Demonstration of the ability to obtain appropriate selectivity over human isoforms and further genetic validation to demonstrate essentiality would help to promote this as a high-priority target.

Farnesyltransferase (Target Under Consideration)

- **Chemical Validation**. Tetrahydroquinoline (THQ) derivatives have been reported as nanomolar inhibitors of recombinant *P. falciparum* protein farnesyltranferase (*Pf*PFT) and *in vitro P. falciparum* asexual blood stage parasites. A THQ analogue showed *in vivo* efficacy in the *P. berghei* mouse model when administered via an osmotic pump.[47] Parasites resistant to tetrahydroquinolines showed mutations at the lipid substate binding site of *Pf*PFT.[48] More recently, MalDA members identified two different mutations in amino acid 515 (A515V and A515T) in parasite clones resistant to MMV019066 (Figure S1), a sub-micromolar inhibitor of *P. falciparum* growth.[49]
- **Genetic Validation**. This gene was found to be essential in the piggyBac insertion mutagenesis and the knockdown screens. Further work is ongoing to genetically validate this target using conditional knockdowns and vulnerability testing.
- **Resistance Potential**. Resistance studies with MMV019066 indicate an MIR of 8.
- **Druggability**. The human orthologue is a well explored target for cancer. Multiple farnesyltransferase inhibitors with drug-like properties have been developed for anticancer therapy.[50] Last year the FDA approved Lonafarnib the first farnesyl transferase inhibitor for the treatment of Hutchison-Gilford progeria syndrome.[51,52]
- **TCP fit**. MMV019066 displays a low rate of kill in the PRR assay. Farnesyltransferase is conserved across *Plasmodium* species.
- **Toxicity**. There are assays to determine selectivity for the *Plasmodium* versus the human enzyme. THQ and other chemotypes have been reported to inhibit selectively *Plasmodium* farnesyltransferase (> 1,000-fold selectivity vs human enzyme).[53]
- **Novelty**. There are no inhibitors of this target in clinical development for malaria.
- **Assay Readiness**. There are several assay platforms for this target including bead-based fluorescence assays, and assays to monitor pyrophosphatase formation.
- **Structural Information**. There is no crystal structure for *Pf* farnesyltransferase, however there are multiple structures of the human orthologue and a homology model for the *Plasmodium* enzyme has been reported.[54]

This target has been investigated from both oncology and malaria perspectives. We believe that further investigation into this target is warranted. Further investigation into the genetic validation and vulnerability to inhibition is required.

Hexose Transporter (PfHT) (Target Under Consideration)

*Pf*HT is responsible for the uptake of glucose (and other hexoses) into the parasite. Glucose metabolism is the main energy source for blood stage *Plasmodium,* and so is likely to be essential for parasite survival.

- **Chemical Validation**. This has been obtained through a glucose analogue[55,56] and compounds identified from phenotypic screening.[57]
- **Genetic Validation**. A conditional knockdown study indicates that the target is essential. The target is also highlighted as vital in the piggyBac screens.[6]
- **Resistance Potential**. The resistance potential of *Pf*HT is still unknown; experiments to determine the MIR are currently ongoing. However, bioinformatic studies suggest that *Pf*HT is the only glucose transporter in the parasite, so there is likely to be no by-pass mechanism or redundancy.
- **Druggability**. Some of the compounds that have been identified as inhibitors[57] are compliant with Lipinski's rules, suggesting that the target is druggable, but this needs further confirmation.
- **TCP fit**. This has still to be fully explored. The hexose transporter is highly conserved across the species. The rate of kill of the parasites upon *Pf*HT inhibition has yet to be determined.
- **Toxicity**. This has yet to be assessed in detail. There exist several human hexose transporters, which could give rise to toxicity if inhibited. Indications are that *Pf*HT is divergent from the human transporters. Some compounds show selectivity for *Pf*HT over some human transporters, although this needs more work.
- **Novelty**. This is novel from a malaria drug discovery perspective.
- **Assay Readiness**. Several assay formats to facilitate the interrogation of *Pf*HT as a drug target have been developed, including an intracellular glucose FRET sensor. It is also possible to recombinantly express the transporter in a variety of cell lines. These assays could also be developed to measure selectivity compared to human hexose transporters.
- **Structural Information**. The structure of *Pf*HT has recently been solved and key structural differences between *Pf*HT and human GLUT's identified, suggesting a possible path for selectivity.[58]

This target is novel and undoubtably there are some challenges to be overcome prior to progressing it. A particular risk here is selectivity compared to homologues in the human host, given the function and widespread distribution of glucose transporters, though recent structural information suggests this may be possible.[69] This is a novel target with a high degree of associated risk, but one which we think should be further investigated.

Niemann-Pick NCR1 (Target Deprioritised)

- **Chemical Validation**.[59] Through a chemical genetics approach, *Pf*NCR1 has been identified as the molecular target of three structurally diverse phenotypically active small molecules: MMV009108, MMV028038, MMV019662.
- **Genetic Validation**.[59] The essentiality of this enzyme has been demonstrated through a variety of methods. Conditional knockdowns show essentiality *in vitro*. PiggyBac insertion screens indicate that the enzyme has a medium likelihood to be essential.[6] Reconstitution of *Pf*NCR1 mutations found in cells lines resistant to the above compounds, via allelic replacement, confirmed they drive resistance.
- **Resistance Potential**. MIR studies suggest a value of 10^6 to 10^7 with compound-specific variation. The corresponding degree of resistance is moderate (3–10× EC_{50}). Parasites resistant to MMV009108, MMV028038 and MMV019662 were achievable through *in vitro* selections. The A1108T mutation was generated in selections with MMV009108; M398I and A1208E from selections with MMV028038; and S490L and F1436I were observed in selections with MMV019662. In each case, selected resistant mutants did not display reduced fitness *in vitro.*
- **Druggability**. Three diverse chemical scaffolds have been shown to target the protein. The three small molecule compounds do not have drug like properties, however there is a chance that they could be modified to become drugs.
- **TCP fit**. This target best aligns with TCP1 because the aforementioned tool compounds are exclusively active against the asexual blood stages of *P. falciparum* (EC_{50} 300–600 nM) and inactive against *P. berghei* liver-stage parasites. Notably, these compounds induce a slow-kill profile which is not an ideal characteristic for malaria treatment.
- **Toxicity**. There is relatively low homology between the *Plasmodium* and human orthologues and good selectivity (SI > 100) has been observed against mammalian cell lines.
- **Novelty**. *Pf*NCR1 is a novel target for malaria.
- **Assay Readiness**. In the absence of a biochemical assay, miniaturized phenotypic screens using the *Pf*NCR1-knockdown line have been developed to sensitize parasites to *Pf*NCR1 inhibitors. As a secondary screen, a saponin-sensitivity assay has been developed to validate the PfNCR1 inhibition phenotype.
- **Structural Information**. A *Plasmodium* structure is not yet available; however, the structures of the yeast[60] and human[61] orthologues are available to support homology modelling.

Although *Pf*NCR1 has been shown, by these data depicted above, to be a valuable, essential and novel target for malaria, recent studies have suggested that this target is susceptible to relatively easy generation of resistance when challenged with inhibitors. Because of this, combined with slow kill and single-stage efficacy, the target has been de-prioritised.

References

1. Alam, M. M.; Sanchez-Azqueta, A.; Janha, O.; Flannery, E. L.; Mahindra, A.; Mapesa, K.; Char, A. B.; Sriranganadane, D.; Brancucci, N. M. B.; Antonova-Koch, Y.; Crouch, K.; Simwela, N. V.; Millar, S. B.; Akinwale, J.; Mitcheson, D.; Solyakov, L.; Dudek, K.; Jones, C.; Zapatero, C.; Doerig, C.; Nwakanma, D. C.; Vázquez, M. J.; Colmenarejo, G.; Lafuente-Monasterio, M. J.; Leon, M. L.; Godoi, P. H. C.; Elkins, J. M.; Waters, A. P.; Jamieson, A. G.; Álvaro, E. F.; Ranford-Cartwright, L. C.; Marti, M.; Winzeler, E. A.; Gamo, F. J.; Tobin, A. B., Validation of the protein kinase PfCLK3 as a multistage cross-species malarial drug target. *Science* **2019,** *365*, eaau1682.

2. Alam, M. M.; Solyakov, L.; Bottrill, A. R.; Flueck, C.; Siddiqui, F. A.; Singh, S.; Mistry, S.; Viskaduraki, M.; Lee, K.; Hopp, C. S.; Chitnis, C. E.; Doerig, C.; Moon, R. W.; Green, J. L.; Holder, A. A.; Baker, D. A.; Tobin, A. B., Phosphoproteomics reveals malaria parasite Protein Kinase G as a signalling hub regulating egress and invasion. *Nat Commun* **2015,** *6*, 7285.

3. McRobert, L.; Taylor, C. J.; Deng, W.; Fivelman, Q. L.; Cummings, R. M.; Polley, S. D.; Billker, O.; Baker, D. A., Gametogenesis in malaria parasites is mediated by the cGMP-dependent protein kinase. *PLoS Biol* **2008,** *6*, e139.

4. Baker, D. A.; Stewart, L. B.; Large, J. M.; Bowyer, P. W.; Ansell, K. H.; Jiménez-Díaz, M. B.; El Bakkouri, M.; Birchall, K.; Dechering, K. J.; Bouloc, N. S.; Coombs, P. J.; Whalley, D.; Harding, D. J.; Smiljanic-Hurley, E.; Wheldon, M. C.; Walker, E. M.; Dessens, J. T.; Lafuente, M. J.; Sanz, L. M.; Gamo, F. J.; Ferrer, S. B.; Hui, R.; Bousema, T.; Angulo-Barturén, I.; Merritt, A. T.; Croft, S. L.; Gutteridge, W. E.; Kettleborough, C. A.; Osborne, S. A., A potent series targeting the malarial cGMP-dependent protein kinase clears infection and blocks transmission. *Nat Commun* **2017,** *8*, 430.

5. Vanaerschot, M.; Murithi, J. M.; Pasaje, C. F. A.; Ghidelli-Disse, S.; Dwomoh, L.; Bird, M.; Spottiswoode, N.; Mittal, N.; Arendse, L. B.; Owen, E. S.; Wicht, K. J.; Siciliano, G.; Bösche, M.; Yeo, T.; Kumar, T. R. S.; Mok, S.; Carpenter, E. F.; Giddins, M. J.; Sanz, O.; Ottilie, S.; Alano, P.; Chibale, K.; Llinás, M.; Uhlemann, A. C.; Delves, M.; Tobin, A. B.; Doerig, C.; Winzeler, E. A.; Lee, M. C. S.; Niles, J. C.; Fidock, D. A., Inhibition of Resistance-Refractory *P. falciparum* Kinase PKG Delivers Prophylactic, Blood Stage, and Transmission-Blocking Antiplasmodial Activity. *Cell Chem Biol* **2020,** *27*, 806-816.e8.

6. Zhang, M.; Wang, C.; Otto, T. D.; Oberstaller, J.; Liao, X.; Adapa, S. R.; Udenze, K.; Bronner, I. F.; Casandra, D.; Mayho, M.; Brown, J.; Li, S.; Swanson, J.; Rayner, J. C.; Jiang, R. H. Y.; Adams, J. H., Uncovering the essential genes of the human malaria parasite *Plasmodium falciparum* by saturation mutagenesis. *Science* **2018,** *360*, eaap7847.

7. Roskoski, R., Jr., Properties of FDA-approved small molecule protein kinase inhibitors: A 2020 update. *Pharmacol Res* **2020,** *152*, 104609.

8. Roskoski, R., Jr., Classification of small molecule protein kinase inhibitors based upon the structures of their drug-enzyme complexes. *Pharmacol Res* **2016,** *103*, 26-48.

9. Zuccotto, F.; Ardini, E.; Casale, E.; Angiolini, M., Through the "gatekeeper door": exploiting the active kinase conformation. *J Med Chem* **2010,** *53*, 2681-2694.

10. Choi, R.; Hulverson, M. A.; Huang, W.; Vidadala, R. S. R.; Whitman, G. R.; Barrett, L. K.; Schaefer, D. A.; Betzer, D. P.; Riggs, M. W.; Doggett, J. S.; Hemphill, A.; Ortega-Mora, L. M.; McCloskey, M. C.; Arnold, S. L. M.; Hackman, R. C.; Marsh, K. C.; Lynch, J. J.; Freiberg, G. M.; Leroy, B. E.; Kempf, D. J.; Choy, R. K. M.; de Hostos, E. L.; Maly, D. J.; Fan, E.; Ojo, K. K.; Van Voorhis, W. C., Bumped Kinase Inhibitors as therapy for apicomplexan parasitic diseases: lessons learned. *Int J Parasitol* **2020,** *50*, 413-422.

11. El Bakkouri, M.; Kouidmi, I.; Wernimont, A. K.; Amani, M.; Hutchinson, A.; Loppnau, P.; Kim, J. J.; Flueck, C.; Walker, J. R.; Seitova, A.; Senisterra, G.; Kakihara, Y.; Kim, C.; Blackman, M. J.; Calmettes, C.; Baker, D. A.; Hui, R., Structures of the cGMP-dependent protein kinase in malaria parasites reveal a unique structural relay mechanism for activation. *Proc Natl Acad Sci U S A* **2019,** *116*, 14164-14173.

12. Davis, M. I.; Hunt, J. P.; Herrgard, S.; Ciceri, P.; Wodicka, L. M.; Pallares, G.; Hocker, M.; Treiber, D. K.; Zarrinkar, P. P., Comprehensive analysis of kinase inhibitor selectivity. *Nat Biotechnol* **2011,** *29*, 1046-1051.
13. Gao, Y.; Davies, S. P.; Augustin, M.; Woodward, A.; Patel, U. A.; Kovelman, R.; Harvey, K. J., A broad activity screen in support of a chemogenomic map for kinase signalling research and drug discovery. *Biochem J* **2013,** *451*, 313-328.
14. Anastassiadis, T.; Deacon, S. W.; Devarajan, K.; Ma, H.; Peterson, J. R., Comprehensive assay of kinase catalytic activity reveals features of kinase inhibitor selectivity. *Nat Biotechnol* **2011,** *29*, 1039-1045.
15. Byun, J. A.; Van, K.; Huang, J.; Henning, P.; Franz, E.; Akimoto, M.; Herberg, F. W.; Kim, C.; Melacini, G., Mechanism of allosteric inhibition in the *Plasmodium falciparum* cGMP-dependent protein kinase. *J Biol Chem* **2020,** *295*, 8480-8491.
16. Franz, E.; Knape, M. J.; Herberg, F. W., cGMP Binding Domain D Mediates a Unique Activation Mechanism in *Plasmodium falciparum* PKG. *ACS Infect Dis* **2018,** *4*, 415-423.
17. Heap, R. E.; Hope, A. G.; Pearson, L. A.; Reyskens, K.; McElroy, S. P.; Hastie, C. J.; Porter, D. W.; Arthur, J. S. C.; Gray, D. W.; Trost, M., Identifying Inhibitors of Inflammation: A Novel High-Throughput MALDI-TOF Screening Assay for Salt-Inducible Kinases (SIKs). *SLAS Discov* **2017,** *22*, 1193-1202.
18. Arendse, L. B.; Wyllie, S.; Chibale, K.; Gilbert, I. H., *Plasmodium* Kinases as Potential Drug Targets for Malaria: Challenges and Opportunities. *ACS Infect Dis* **2021,** *7*, 518-534.
19. Kim, S. H.; Bae, S.; Song, M., Recent Development of Aminoacyl-tRNA Synthetase Inhibitors for Human Diseases: A Future Perspective. *Biomolecules* **2020,** *10*, 1625.
20. Herman, J. D.; Pepper, L. R.; Cortese, J. F.; Estiu, G.; Galinsky, K.; Zuzarte-Luis, V.; Derbyshire, E. R.; Ribacke, U.; Lukens, A. K.; Santos, S. A.; Patel, V.; Clish, C. B.; Sullivan, W. J., Jr.; Zhou, H.; Bopp, S. E.; Schimmel, P.; Lindquist, S.; Clardy, J.; Mota, M. M.; Keller, T. L.; Whitman, M.; Wiest, O.; Wirth, D. F.; Mazitschek, R., The cytoplasmic prolyl-tRNA synthetase of the malaria parasite is a dual-stage target of febrifugine and its analogs. *Sci Transl Med* **2015,** *7*, 288ra77.
21. Herman, J. D.; Rice, D. P.; Ribacke, U.; Silterra, J.; Deik, A. A.; Moss, E. L.; Broadbent, K. M.; Neafsey, D. E.; Desai, M. M.; Clish, C. B.; Mazitschek, R.; Wirth, D. F., A genomic and evolutionary approach reveals non-genetic drug resistance in malaria. *Genome Biol* **2014,** *15*, 511.
22. Zhou, H.; Sun, L.; Yang, X. L.; Schimmel, P., ATP-directed capture of bioactive herbal-based medicine on human tRNA synthetase. *Nature* **2013,** *494*, 121-124.
23. Jain, V.; Yogavel, M.; Kikuchi, H.; Oshima, Y.; Hariguchi, N.; Matsumoto, M.; Goel, P.; Touquet, B.; Jumani, R. S.; Tacchini-Cottier, F.; Harlos, K.; Huston, C. D.; Hakimi, M. A.; Sharma, A., Targeting Prolyl-tRNA Synthetase to Accelerate Drug Discovery against Malaria, Leishmaniasis, Toxoplasmosis, Cryptosporidiosis, and Coccidiosis. *Structure* **2017,** *25*, 1495-1505.e6.
24. Manickam, Y.; Chaturvedi, R.; Babbar, P.; Malhotra, N.; Jain, V.; Sharma, A., Drug targeting of one or more aminoacyl-tRNA synthetase in the malaria parasite *Plasmodium falciparum*. *Drug Discov Today* **2018,** *23*, 1233-1240.
25. Seiradake, E.; Mao, W.; Hernandez, V.; Baker, S. J.; Plattner, J. J.; Alley, M. R.; Cusack, S., Crystal structures of the human and fungal cytosolic Leucyl-tRNA synthetase editing domains: A structural basis for the rational design of antifungal benzoxaboroles. *J Mol Biol* **2009,** *390*, 196-207.
26. Hewitt, S. N.; Dranow, D. M.; Horst, B. G.; Abendroth, J. A.; Forte, B.; Hallyburton, I.; Jansen, C.; Baragana, B.; Choi, R.; Rivas, K. L.; Hulverson, M. A.; Dumais, M.; Edwards, T. E.; Lorimer, D. D.; Fairlamb, A. H.; Gray, D. W.; Read, K. D.; Lehane, A. M.; Kirk, K.; Myler, P. J.; Wernimont, A.; Walpole, C.; Stacy, R.; Barrett, L. K.; Gilbert, I. H.; Van Voorhis, W. C., Biochemical and Structural Characterization of Selective Allosteric Inhibitors of the *Plasmodium falciparum* Drug Target, Prolyl-tRNA-synthetase. *ACS Infect Dis* **2017,** *3*, 34-44.

27. Cestari, I.; Stuart, K., A spectrophotometric assay for quantitative measurement of aminoacyl-tRNA synthetase activity. *J Biomol Screen* **2013,** *18*, 490-497.
28. Kato, N.; Comer, E.; Sakata-Kato, T.; Sharma, A.; Sharma, M.; Maetani, M.; Bastien, J.; Brancucci, N. M.; Bittker, J. A.; Corey, V., Diversity-oriented synthesis yields novel multistage antimalarial inhibitors. *Nature* **2016,** *538*, 344-349.
29. Ding, X. C.; Ubben, D.; Wells, T. N., A framework for assessing the risk of resistance for anti-malarials in development. *Malar. J.* **2012,** *11*, 292.
30. Sharma, M.; Malhotra, N.; Yogavel, M.; Harlos, K.; Melillo, B.; Comer, E.; Gonse, A.; Parvez, S.; Mitasev, B.; Fang, F. G.; Schreiber, S. L.; Sharma, A., Structural basis of malaria parasite phenylalanine tRNA-synthetase inhibition by bicyclic azetidines. *Nat Commun* **2021,** *12*, 343.
31. Wang, H.; Xu, M.; Engelhart, C. A.; Zhang, X.; Yan, B.; Pan, M.; Xu, Y.; Fan, S.; Liu, R.; Xu, L.; Hua, L.; Schnappinger, D.; Chen, S., Rediscovery of PF-3845 as a new chemical scaffold inhibiting phenylalanyl-tRNA synthetase in *Mycobacterium tuberculosis*. *J Biol Chem* **2021,** *296*, 100257.
32. Taipale, M.; Jarosz, D. F.; Lindquist, S., HSP90 at the hub of protein homeostasis: emerging mechanistic insights. *Nat Rev Mol Cell Biol* **2010,** *11*, 515-528.
33. Acharya, P.; Kumar, R.; Tatu, U., Chaperoning a cellular upheaval in malaria: heat shock proteins in *Plasmodium falciparum*. *Mol Biochem Parasitol* **2007,** *153*, 85-94.
34. Shahinas, D.; Folefoc, A.; Pillai, D. R., Targeting *Plasmodium falciparum* Hsp90: Towards Reversing Antimalarial Resistance. *Pathogens* **2013,** *2*, 33-54.
35. Posfai, D.; Eubanks, A. L.; Keim, A. I.; Lu, K. Y.; Wang, G. Z.; Hughes, P. F.; Kato, N.; Haystead, T. A.; Derbyshire, E. R., Identification of Hsp90 Inhibitors with Anti-*Plasmodium* Activity. *Antimicrob Agents Chemother* **2018,** *62*, e01799-17.
36. Sanchez, J.; Carter, T. R.; Cohen, M. S.; Blagg, B. S. J., Old and New Approaches to Target the Hsp90 Chaperone. *Curr Cancer Drug Targets* **2020,** *20*, 253-270.
37. Banumathy, G.; Singh, V.; Pavithra, S. R.; Tatu, U., Heat shock protein 90 function is essential for *Plasmodium falciparum* growth in human erythrocytes. *J Biol Chem* **2003,** *278*, 18336-18345.
38. Peterson, L. B.; Eskew, J. D.; Vielhauer, G. A.; Blagg, B. S., The hERG channel is dependent upon the Hsp90α isoform for maturation and trafficking. *Mol Pharm* **2012,** *9*, 1841-1846.
39. Ernst, J. T.; Liu, M.; Zuccola, H.; Neubert, T.; Beaumont, K.; Turnbull, A.; Kallel, A.; Vought, B.; Stamos, D., Correlation between chemotype-dependent binding conformations of HSP90α/β and isoform selectivity-Implications for the structure-based design of HSP90α/β selective inhibitors for treating neurodegenerative diseases. *Bioorg Med Chem Lett* **2014,** *24*, 204-208.
40. Kumar, R.; Musiyenko, A.; Barik, S., The heat shock protein 90 of *Plasmodium falciparum* and antimalarial activity of its inhibitor, geldanamycin. *Malar J* **2003,** *2*, 30.
41. Shahinas, D.; Folefoc, A.; Taldone, T.; Chiosis, G.; Crandall, I.; Pillai, D. R., A purine analog synergizes with chloroquine (CQ) by targeting *Plasmodium falciparum* Hsp90 (PfHsp90). *PLoS One* **2013,** *8*, e75446.
42. McLaughlin, S. H.; Ventouras, L. A.; Lobbezoo, B.; Jackson, S. E., Independent ATPase activity of Hsp90 subunits creates a flexible assembly platform. *J Mol Biol* **2004,** *344*, 813-826.
43. Schumacher, R. J.; Hurst, R.; Sullivan, W. P.; McMahon, N. J.; Toft, D. O.; Matts, R. L., ATP-dependent chaperoning activity of reticulocyte lysate. *J Biol Chem* **1994,** *269*, 9493-9499.
44. Sadikot, T.; Swink, M.; Eskew, J. D.; Brown, D.; Zhao, H.; Kusuma, B. R.; Rajewski, R. A.; Blagg, B. S.; Matts, R. L.; Holzbeierlein, J. M.; Vielhauer, G. A., Development of a high-throughput screening cancer cell-based luciferase refolding assay for identifying Hsp90 inhibitors. *Assay Drug Dev Technol* **2013,** *11*, 478-488.
45. Banerjee, M.; Hatial, I.; Keegan, B. M.; Blagg, B. S. J., Assay design and development strategies for finding Hsp90 inhibitors and their role in human diseases. *Pharmacol Ther* **2020,** *221*, 107747.

46. Corbett, K. D.; Berger, J. M., Structure of the ATP-binding domain of *Plasmodium falciparum* Hsp90. *Proteins* **2010,** *78*, 2738-2744.

47. Nallan, L.; Bauer, K. D.; Bendale, P.; Rivas, K.; Yokoyama, K.; Hornéy, C. P.; Pendyala, P. R.; Floyd, D.; Lombardo, L. J.; Williams, D. K.; Hamilton, A.; Sebti, S.; Windsor, W. T.; Weber, P. C.; Buckner, F. S.; Chakrabarti, D.; Gelb, M. H.; Van Voorhis, W. C., Protein Farnesyltransferase Inhibitors Exhibit Potent Antimalarial Activity. *J Med Chem* **2005,** *48*, 3704-3713.

48. Eastman, R. T.; White, J.; Hucke, O.; Yokoyama, K.; Verlinde, C. L.; Hast, M. A.; Beese, L. S.; Gelb, M. H.; Rathod, P. K.; Van Voorhis, W. C., Resistance mutations at the lipid substrate binding site of *Plasmodium falciparum* protein farnesyltransferase. *Mol Biochem Parasitol* **2007,** *152*, 66-71.

49. Cowell, A. N.; Istvan, E. S.; Lukens, A. K.; Gomez-Lorenzo, M. G.; Vanaerschot, M.; Sakata-Kato, T.; Flannery, E. L.; Magistrado, P.; Owen, E.; Abraham, M.; LaMonte, G.; Painter, H. J.; Williams, R. M.; Franco, V.; Linares, M.; Arriaga, I.; Bopp, S.; Corey, V. C.; Gnadig, N. F.; Coburn-Flynn, O.; Reimer, C.; Gupta, P.; Murithi, J. M.; Moura, P. A.; Fuchs, O.; Sasaki, E.; Kim, S. W.; Teng, C. H.; Wang, L. T.; Akidil, A.; Adjalley, S.; Willis, P. A.; Siegel, D.; Tanaseichuk, O.; Zhong, Y.; Zhou, Y.; Llinas, M.; Ottilie, S.; Gamo, F. J.; Lee, M. C. S.; Goldberg, D. E.; Fidock, D. A.; Wirth, D. F.; Winzeler, E. A., Mapping the malaria parasite druggable genome by using in vitro evolution and chemogenomics. *Science* **2018,** *359*, 191-199.

50. Haluska, P.; Dy, G. K.; Adjei, A. A., Farnesyl transferase inhibitors as anticancer agents. *Eur J Cancer* **2002,** *38*, 1685-1700.

51. Ullrich, N. J.; Kieran, M. W.; Miller, D. T.; Gordon, L. B.; Cho, Y. J.; Silvera, V. M.; Giobbie-Hurder, A.; Neuberg, D.; Kleinman, M. E., Neurologic features of Hutchinson-Gilford progeria syndrome after lonafarnib treatment. *Neurology* **2013,** *81*, 427-430.

52. Mullard, A., The FDA approves a first farnesyltransferase inhibitor. *Nat Rev Drug Discov* **2021,** *20*, 8.

53. Alexander, H. K.; MacLean, R. C., Stochastic bacterial population dynamics restrict the establishment of antibiotic resistance from single cells. **2020,** *117*, 19455-19464.

54. Hast, M. A.; Fletcher, S.; Cummings, C. G.; Pusateri, E. E.; Blaskovich, M. A.; Rivas, K.; Gelb, M. H.; Van Voorhis, W. C.; Sebti, S. M.; Hamilton, A. D.; Beese, L. S., Structural basis for binding and selectivity of antimalarial and anticancer ethylenediamine inhibitors to protein farnesyltransferase. *Chem Biol* **2009,** *16*, 181-192.

55. Joët, T.; Eckstein-Ludwig, U.; Morin, C.; Krishna, S., Validation of the hexose transporter of *Plasmodium falciparum* as a novel drug target. *Proc. Natl. Acad. Sci. USA* **2003,** *100*, 7476-7479.

56. Slavic, K.; Delves, M. J.; Prudêncio, M.; Talman, A. M.; Straschil, U.; Derbyshire, E. T.; Xu, Z.; Sinden, R. E.; Mota, M. M.; Morin, C.; Tewari, R.; Krishna, S.; Staines, H. M., Use of a Selective Inhibitor To Define the Chemotherapeutic Potential of the *Plasmodial* Hexose Transporter in Different Stages of the Parasite's Life Cycle. *Antimicrob. Agents Chemother.* **2011,** *55*, 2824-2830.

57. Heitmeier, M. R.; Hresko, R. C.; Edwards, R. L.; Prinsen, M. J.; Ilagan, M. X. G.; Odom John, A. R.; Hruz, P. W., Identification of druggable small molecule antagonists of the *Plasmodium falciparum* hexose transporter PfHT and assessment of ligand access to the glucose permeation pathway via FLAG-mediated protein engineering. *PLoS One* **2019,** *14*, e0216457.

58. Qureshi, A. A.; Suades, A.; Matsuoka, R.; Brock, J.; McComas, S. E.; Nji, E.; Orellana, L.; Claesson, M.; Delemotte, L.; Drew, D., The molecular basis for sugar import in malaria parasites. *Nature* **2020,** *578*, 321-325.

59. Istvan, E. S.; Das, S.; Bhatnagar, S.; Beck, J. R.; Owen, E.; Llinas, M.; Ganesan, S. M.; Niles, J. C.; Winzeler, E.; Vaidya, A. B.; Goldberg, D. E., *Plasmodium* Niemann-Pick type C1-related protein is a druggable target required for parasite membrane homeostasis. *Elife* **2019,** *8*, e40529.

60. Winkler, M. B. L.; Kidmose, R. T.; Szomek, M.; Thaysen, K.; Rawson, S.; Muench, S. P.; Wüstner, D.; Pedersen, B. P., Structural Insight into Eukaryotic Sterol Transport through Niemann-Pick Type C Proteins. *Cell* **2019,** *179*, 485-497.e18.

61. Gong, X.; Qian, H.; Zhou, X.; Wu, J.; Wan, T.; Cao, P.; Huang, W.; Zhao, X.; Wang, X.; Wang, P.; Shi, Y.; Gao, G. F.; Zhou, Q.; Yan, N., Structural Insights into the Niemann-Pick C1 (NPC1)-Mediated Cholesterol Transfer and Ebola Infection. *Cell* **2016,** *165*, 1467-1478.
